# Pose Stabilization Control for Base of Combined System Using Feedforward Compensation PD Control During Target Satellite Transposition

**DOI:** 10.3390/s26010206

**Published:** 2025-12-28

**Authors:** Zhonghua Hu, Jinlong Yang, Wenfu Xu, Hengtai Chen, Longkun Xu, Deshan Meng

**Affiliations:** 1 School of Mechanical & Automotive Engineering, Liaocheng University, Liaocheng 252000, China; yjl_lcu@163.com (J.Y.); cht_lcu@163.com (H.C.); xulongkun@lcu.edu.cn (L.X.); 2 School of Mechanical Engineering and Automation, Harbin Institute of Technology, Shenzhen 518055, China; wfxu@hit.edu.cn; 3 School of Aeronautics and Astronautics, Shenzhen Campus, Sun Yat-sen University, Shenzhen 518107, China

**Keywords:** space robot–target satellite combined system, target satellite transposition, dynamics modeling, pose stabilization control

## Abstract

During the transposition of a target satellite, dynamic coupling between the target satellite, the manipulators, and the base frequently leads to disturbances in the base’s attitude. To deal with the issue, this paper proposed a pose stabilization method for the base of the post-capture combined system using the feedforward compensation PD control. Firstly, the mission sequence for repositioning a target satellite using a discrete-serpentine heterogeneous dual-arm space robot (DSHDASR) was analyzed. The dynamics model of the combined system, composed of the DSHDASR and a target satellite, was established based on the Newton–Euler recursive formulation. Then, the pose stabilization method integrating dynamic feedforward compensation and PD control was developed to stabilize the base of the combined system. Finally, the mission of target satellite transposition was simulated through the co-simulation model. Compared with the traditional control algorithms, the position accuracy and attitude accuracy for the proposed method showed an overall improvement. The results demonstrated that the proposed method significantly reduced base pose errors under high-load and disturbed conditions.

## 1. Introduction

Space robotic manipulators are essential for on-orbit servicing (OOS) [1,2], including debris removal, structure assembly, and satellite repair [3]. Based on the number of joints and link characteristics, space manipulators can be categorized into three types: discrete arms [4], serpentine arms [5], and continuum arms [6]. Unlike continuum arms, the discrete and serpentine manipulators present fewer challenges in terms of accurate modeling and precise control. Therefore, they are most widely adopted to carry out OOS. Furthermore, compared to a single discrete or serpentine arm, the DSHDASR combines the advantages of both types of arms, which ultimately overcomes specific limitations of individual manipulators [7]. Before conducting OOS, the space robotic arms installed on the DSHDASR should grasp the target satellite firstly, and then transpose it from the current location to the desired position for the subsequent operations [8]. Once the target satellite has been captured, the combined system is formed. However, during the target satellite transposition, strong dynamic coupling between the target, the manipulators, and the base can disturb the base’s pose (position and attitude). These disturbances not only reduce the tracking accuracy of manipulators but also bring potential risks to subsequent operations [9]. To validate space robotic control strategies under near-realistic microgravity conditions, various ground-based microgravity emulation platforms have been developed in recent years [10,11]. Therefore, suppressing these disturbances poses a great challenge for achieving precise tracking during target transposition, particularly under the condition of strong dynamic coupling.

To address this challenge, considerable research focused on pose stabilization control. Papadopoulos and Dubowsky [12] derived the dynamics model of space robotic manipulators using a Lagrangian framework, enabling coordinated control between the manipulator and the base. To achieve the desired attitude transposition, Yamada [13] proposed a joint path-planning method to achieve a desired change in attitude. By combining the Backstepping method and the radial basis function neural networks, Zhan et al. [14] designed an adaptive controller to ensure the trajectory tracking and base stabilization. Taking the end-effector as a Virtual Base, Zong et al. [15] established a new dynamics model of free-floating space manipulators to accomplish end-effector trajectory tracking and minimum disturbances to the base. Subsequently, Gong et al. [16] developed a dynamics model with parametric uncertainties to deal with the reactionless control of pose tracking. Based on the extended Jacobian model, Xu [17] designed a passivity adaptive controller to achieve the pose attitude adjustment and trajectory tracking. Considering the rigid body modes, Kuck et al. [18] developed a benchmark feedback controller to minimize trajectory tracking errors. While aforementioned methods can achieve base pose control, they do not account for the presence of a payload on the arm’s end-effector.

A payload significantly intensifies the dynamic coupling between the manipulators and the base. This brings a significant challenge to base stabilization control. To deal with the problem, Raina et al. [19] proposed a unified framework for impact dynamics modeling and post-capture reactionless control to guarantee attitude stabilization of the base. Wang et al. [20] developed a coordinated control method for post-capture operations, enabling a redundant space robot to maintain the attitude stability of the combined system. It should be noted that in both the methods above, the payload-to-base mass ratio is under 10%. For high proportion payloads, Chang et al. [21] presented an adaptive Backstepping-based nonsingular fast integral terminal sliding mode control method for the attitude stabilization of space robots after capturing a tumbling target. Woodward et al. [22] designed a Lyapunov-based control law to drive the base’s angular velocity attitude error asymptotically to zero. Based on dynamic coupling control with an iterative extended Kalman filter, Zhang et al. [23] proposed a rapid attitude stabilization strategy for highly robust base-attitude stabilization. Huang et al. [24] developed a robust attitude control method based on a disturbance observer and dynamic control allocation for the post-capture attitude control of the combined system, which ensured the prescribed attitude performance under uncertainties and external disturbances. Song et al. [25] applied a model-free control method for the attitude and orbital control of a post-capture combined spacecraft. Although these methods address base stabilization with large payloads on the end-effector, they do not consider the end-effector’s trajectory tracking performance.

Recently, the rapid development of intelligent algorithms has led to their application in base stabilization control. Liu et al. [26] investigated a trajectory planning and coordination control method to minimize the target’s attitude and disturbances to the base using the multi-objective particle swarm optimization (MOPSO) algorithm. Based on the deep Koopman operator-based modeling approach with model predictive control, Reference [27] achieved high-precision joint tracking and effective suppression of base attitude disturbances. According to reinforcement learning, Wang et al. [28] and Dong et al. [29] proposed a multi-target trajectory optimization method and a sequential optimization approach, respectively, to control the base’s dynamic response and stabilize its attitude. In addition, Ma et al. [30] applied deep reinforcement learning to restore a satellite’s attitude, which is a task analogous to base attitude control.

As discussed above, although numerous methods for base pose stabilization have been proposed, each method has certain limitations. Some approaches established the relationship between base pose disturbance and manipulator motion to achieve stabilization control of the base, yet did not account for the case of a payload on the end-effector. Other methods addressed base stabilization with a payload, but they did not account for end-effector pose tracking. Furthermore, while some intelligent algorithms could effectively handle base pose stabilization, their computational demands often made them impractical for real-time control on spacecraft hardware. To tackle these issues, a pose stabilization method based on feedforward compensation PD control was proposed for the base of the combined system, while the DSHDASR was transposing the target satellite from the initial pose to the desired pose. Compared with the PD control method and Backstepping method, this method introduces the reaction force and torque acting on the base in the control law, effectively decreasing the strong dynamic coupling effects on the base. In addition, compared with existing approaches based on adaptive schemes or intelligent algorithms, the proposed method has advantages including a clear computational structure, low computational complexity, and high computational efficiency. This feature makes the proposed strategy more suitable for spacecraft platforms within limited onboard computational resources and offers favorable potential for real-time implementation.

The remainder of this paper is organized as follows. Section 2 analyzes the overall process of the target satellite transposition. In Section 3, the dynamics model of the combined system is established by the recursive Newton–Euler formulation. Based on the PD control with feedforward compensation, Section 4 proposes the pose stabilization method for the base during the target satellite transposition. In Section 5, the proposed method is verified through the comparison simulations including the proposed method, the traditional PD method, and the Backstepping method. A comprehensive discussion of the challenges and simplifications is given in Section 6. Based on the analysis of previous sections, the last section summarizes the work of this study.

## 2. Overall Task Description

**Figure 1:**
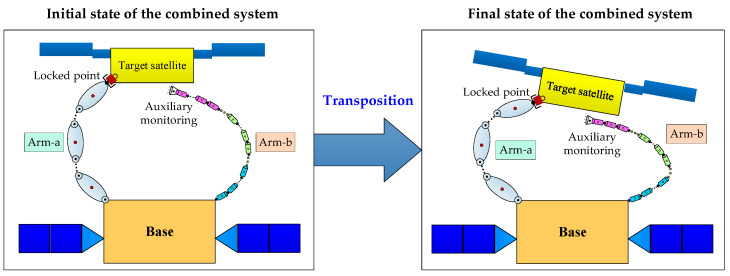
The illustration for target satellite transposition.

After capture, the target satellite often must be moved to a suitable location for subsequent on-orbit operations. This movement process is known as the target satellite transposition, as shown in Figure 1. At the initial state, the target satellite has been captured by the DSHDASR. The DSHDASR is composed of a base, a non-offset discrete arm (namely Arm-a) and a segmented cable-driven hyper-redundant serpentine arm (namely Arm-b). Together, the DSHDASR and the target satellite form the combined system. During transposition, Arm-a serves as the primary manipulator for moving the target satellite from the initial pose to the final pose (i.e., desired position and attitude) along a predefined trajectory, while Arm-b is used as the auxiliary manipulator, providing visual monitoring of Arm-a’s operation. Due to dynamic coupling between the base and the two manipulators, the base moves as Arm-a and Arm-b execute the coordinated transposition. As a result, the motion of the base alters the end-effector trajectories of both arms.

To ensure that the end-effectors of the two arms follow their predefined trajectories, it is necessary to maintain the base in a stable state. Therefore, this paper investigates control for stabilizing the base of the combined system during target satellite transposition. Some assumptions are given as follows.

(1) The mass and geometric parameters of the target satellite and the DSHDASR are assumed to be known and are considered to be exact values in this study.
(2) The base pose control can be achieved by thrusters and reaction wheels, whose installation positions and directions are known.
(3) The target satellite and the end-effector of the non-offset discrete arm remain in a securely locked state during the transposing process.
(4) Joint friction is neglected in the dynamic modeling and control design, and all joints are assumed to be ideal frictionless revolute joints.
(5) The structural flexibility of the links and joints for the DSHDASR, as well as mechanical backlash and other non-ideal effects, are not taken into account.

## 3. Dynamics Modeling of the Combined System

**Figure 2:**
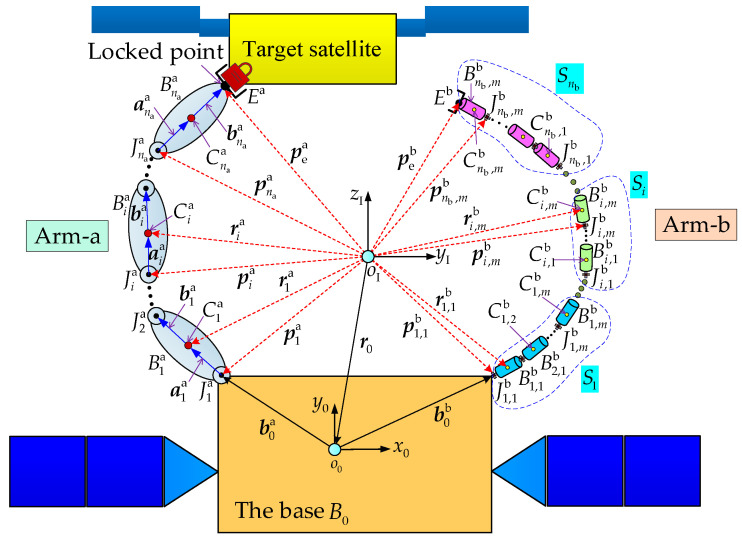
The generalized model of the combined system.

As shown in Figure 2, the combined system is formed after the target satellite is captured by the DSHDASR.

As discussed above, the DSHDASR consists of a base, a non-offset discrete arm (Arm-a), and a segmented cable-driven serpentine arm (Arm-b). For Arm-a, each joint only has one rotational axis that is driven by a motor. The total number of joints is denoted by $n_{a}$. In contrast, Arm-b is composed of $n_{b}$ segments with identical mechanical structures. Each segment has *m* associated universal joints, which are driven by several cables. An associated universal joint possesses a Pitch-axis and a Yaw-axis. For each segment, all associated universal joints are hybrid active–passive joints [31]. Specifically, the first universal joint in a segment is active and follows planned values, while the remaining joints are passive and move synchronously with it. {*x*_I_, *y*_I_, *z*_I_} and {*x*_0_, *y*_0_, *z*_0_} are the inertial frame and the frame fixed on the base center of mass, respectively. Unless otherwise stated, all the variables are represented in the inertial frame.

The Newton–Euler formulation is a well-known method that offers advantages in computational efficiency and numerical stability, making it suitable for real-time control. As a result, the Newton–Euler formulation is adopted to establish the dynamic model of the combined system.

### 3.1. Forward Recursion for Velocities and Accelerations Computation

As shown in Figure 2, $J_{i}^{a}$ and $B_{i}^{a}$ represent the *i*th joint and link of Arm-a. $C_{i}^{a}$ is the center of mass of $B_{i}^{a}$ and its position vector is denoted by $r_{i}^{a}$. $a_{i}^{a}$ represents the vector from the joint $J_{i}^{a}$ to the center $C_{i}^{a}$ and $b_{i}^{a}$ is the vector from the center $C_{i}^{a}$ to the joint $J_{i+1}^{a}$. For the *i*th joint of Arm-a, the position vector of $J_{i}^{a}$ is obtained as follows:

(1) $$p_{i}^{a}=r_{0}+b_{0}^{a}+\sum_{j=1}^{i-1}\left(a_{j}^{a}+b_{j}^{a}\right)\in R^{3\times 1}$$

where $r_{0}$ is the position vector of the center of mass for the base. $b_{0}^{a}$ denotes the vector from the base’s center of mass to the first joint of Arm-a. According to the principle of relative motion, the angular velocity and the linear velocity of $J_{i}^{a}$ expressed in the *i*th joint frame are obtained as follows:

(2) ωiai=Ri−1aiωi−1ai−1+kiai·q˙iaviai=Ri−1aivi−1ai−1+ωi−1ai−1×ai−1ai−1+bi−1ai−1

where Ri−1ai stands for the transformation matrix from the (*i* − 1)th joint frame to the *i*th joint frame for Arm-a. Regarding the other parameters in Equation (2), the left superscript means that the vector is described in the frame indicated by the superscript, i.e., vi−1ai−1, ωi−1ai−1, ai−1ai−1, and bi−1ai−1 are all expressed in the (*i* − 1)th joint frame of Arm-a. The subsequent notations follow the same convention. kiai and ${\dot{q}}_{j}^{a}$ refer to the rotation axis and the angular velocity magnitude of the *i*th joint for Arm-a, respectively.

Differentiating Equation (2), the angular acceleration and linear acceleration of $J_{i}^{a}$ are derived as follows:

(3) ω˙iai=Ri−1aiω˙i−1ai−1+Ri−1aiωi−1ai−1×kiai·q˙ia+kiai·q¨iav˙iai=Ri−1aiv˙i−1ai−1+ω˙i−1ai−1×ai−1ai−1+bi−1ai−1+ωi−1ai−1×ωi−1ai−1×ai−1ai−1+bi−1ai−1

where ${\ddot{q}}_{i}^{a}$ denotes the angular acceleration magnitude of $J_{i}^{a}$.

Based on Equation (3), the angular and linear accelerations of $C_{i}^{a}$ are obtained as follows.

(4) ω˙Ciai=ω˙iai=Ri−1aiω˙i−1ai−1+Ri−1aiωi−1ai−1×kiai·q˙ia+kiai·q¨iav˙Ciai=v˙iai+ω˙Ciai×aiai+ωiai×ωiai×aiai

For Arm-b, $J_{i,j}^{b}$ and $B_{i,j}^{b}$ are the *j*th joint and the *j*th link in the *i*th segment. $C_{i,j}^{b}$ refers the center of mass of $B_{i,j}^{b}$ and its position vector is named by $r_{i,j}^{b}$. The position vector of the *j*th joint in the *i*th segment of Arm-b can be calculated as follows:

(5) $$p_{i,j}^{b}=r_{0}+b_{0}^{b}+\sum_{h=1}^{i-1}\sum_{n=1}^{m}\left(a_{h,n}^{b}+b_{h,n}^{b}\right)+\sum_{n=1}^{j-1}\left(a_{i,n}^{b}+b_{i,n}^{b}\right)\in R^{3\times 1}$$

where $b_{0}^{b}$ is the vector from the base’s center of mass to the first joint of Arm-b. $a_{i,j}^{b}$ represents the vector from the joint $J_{i,j}^{b}$ to the center $C_{i,j}^{b}$. $b_{i,j}^{b}$ is the vector from the center $C_{i,j}^{b}$ to the joint $J_{i,j+1}^{b}$.

For the *j*th joint within the *i*th segment of Arm-b, its two rotational axes are denoted as $k_{i,\;j,1}^{b}$ and $k_{i,\;j,2}^{b}$, and the corresponding angular velocities are ${\dot{q}}_{i,\;j,1}^{b}$ and ${\dot{q}}_{i,\;j,2}^{b}$. Since the universal joints in the same segment are hybrid active and passive, the movement law of universal joints in the *i*th segment can be expressed as follows.

(6) $$\left\{\begin{array}{l} {\dot{q}}_{i,j,1}^{b}={\dot{q}}_{i,j+1,2}^{b}\\ {\dot{q}}_{i,j,2}^{b}={\dot{q}}_{i,j+1,1}^{b} \end{array}\right.\;\;\;\;\;i=1,2,\ldots ,n_{b};\;\;\;\;\;j=1,2,\ldots ,m-1$$

Based on the kinematics of relative motion, the angular velocity and the linear velocity of the *j*th joint in the *i*th segment are obtained as follows:

(7) ωi,jbi,j,2=Ri,j−1,2bi,j,2ωi,j−1bi,j−1,2+Ri,j,1bi,j,2ki,j,1bi,j,1·q˙i,j,1b+ki,j,2bi,j,2·q˙i,j,2bvi,jbi,j,2=Ri,j−1,2bi,j,2vi,j−1bi,j−1,2+ωi,j−1bi,j−1,2×ai,j−1bi,j−1,2+bi,j−1bi,j−1,2

where Ri,j−1,2bi,j,2 is the transformation matrix from the second rotational axis of the (*j* − 1)th joint to the *j*th joint in the *i*th segment. With respect to the remaining parameters, the left superscript represents that the vector is expressed in the frame attached to the second rotational axis of the (*j* − 1)th joint in the *i*th segment. ki,j,1bi,j,1 and ki,j,2bi,j,2 are the first and second rotational axes of the *j*th joint. Their corresponding angular velocities are ${\dot{q}}_{i,j,1}^{b}$ and ${\dot{q}}_{i,j,2}^{b}$, respectively.

Differentiating Equation (7), the angular and linear accelerations of $J_{i,j}^{b}$ are computed as follows.

(8) ω˙i,jbi,j,2=Ri,j−1,2bi,j,2ωi,j−1bi,j−1,2×Ri,j,1bi,j,2zi,j,1bi,j,1·q˙i,j,1b+zi,j,2bi,j,2·q˙i,j,2b             +Ri,j,1bi,j,2zi,j,1bi,j,1·q˙i,j,1b×zi,j,2bi,j,2·q˙i,j,2b+Ri,j,1bi,j,2zi,j,1bi,j,1·q¨i,j,1b             +zi,j,2bi,j,2·q¨i,j,2b+Ri,j−1,2bi,j,2ω˙i,j−1bi,j−1,2v˙i,jbi,j,2=Ri,j−1,2bi,j,2·v˙i,j−1bi,j−1,2+ω˙i,j−1bi,j−1,2×ai,j−1bi,j−1,2+bi,j−1bi,j−1,2+ωi,j−1bi,j−1,2×ωi,j−1bi,j−1,2×ai,j−1bi,j−1,2+bi,j−1bi,j−1,2

According to Equation (8), the angular acceleration and linear acceleration of $C_{i,j}^{b}$ can be obtained as follows.

(9) ω˙Ci,jbi,j,2=ω˙i,jbi,j,2v˙Ci,jbi,j,2=v˙i,jbi,j,2+ω˙i,jbi,j,2×ai,jbi,j,2+ωi,jbi,j,2×ωi,jbi,j,2×ai,jbi,j,2

### 3.2. Backward Recursion for Joint Forces and Torques Calculation

The base’s motion including angular velocity, angular acceleration, linear velocity, and linear acceleration can be obtained from measurement by gyroscopes, accelerometers, or other sensors. In other words, ω0a0, ω˙0a0, v0a0, and v˙0a0 are known. On the basis of Equations (4) and (9), the angular and linear accelerations of each link’s center of mass for Arm-a and Arm-b can be determined.

**Figure 3:**
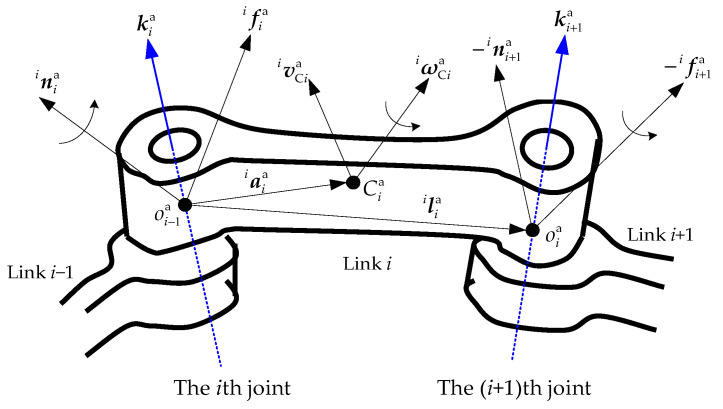
The force analysis for the *i*th link of Arm-a.

For both arms, joint forces and torques are calculated in reverse order, propagating from the end-effector back toward the base. For Arm-a, the dynamic relationship of the *i*th link is shown in Figure 3.

The gravitational force acting on each link of Arm-a can be considered as zero, due to the microgravity environment where the arm is. For the *i*th link of Arm-a, the force and torque balance equations are derived as follows.

(10) fCiai−fiai−fi+1ai=0nCiai−niai−ni+1ai+fiai×aiai−fi+1ai×aiai−liai=0

where fCiai and nCiai are the inertia force and torque of the *i*th link. fiai and niai refer to the force and torque exerted by the (*i* − 1)th link on the *i*th link. fi+1ai and ni+1ai are the force and torque exerted by the *i*th link on the (*i* + 1)th link. liai is the position vector from the *i*th joint to the (*i* + 1)th joint. They are all expressed in the frame fixed on the *i*th joint.

According to Newton’s laws and Euler’s equations of motion, the inertia force and torque of the *i*th link can be obtained as follows:

(11) fCiai=mia·v˙CiainCiai=Iiai·ω˙Ciai+ωCiai×Iiai·ωCiai

where $m_{i}^{a}$ and Iiai are the mass and inertia tensor of the *i*th link.

Substituting Equation (11) into Equation (10), the relationship between the motion and the applied force and torque for the *i*th link can be obtained as follows.

(12) fiai−fi+1ai=mia·v˙Ciainiai−ni+1ai+fiai×aiai−fi+1ai×aiai−liai=Iiai·ω˙Ciai+ωCiai×Iiai·ωCiai

Here, ${\dot{v}}_{Ci}^{a}$ and ω˙Ciai have been calculated based on Equation (4). Substituting Equation (4) into Equation (12), the following equations hold.

(13) fiai=Ri+1aifi+1ai+1+miav˙iai+ω˙Ciai×rCiai+ωiai×ωiai×rCiainiai=Ri+1aini+1ai+1−fiai×aiai+fi+1ai×aiai−liai+Iiai·ω˙Ciai+ωCiai×Iiai·ωCiai

Notably, the issue of the virtual link $n_{a}+1$ has occurred during the backward recursion for calculating forces and torques. To address this, the external environment of Arm-a is treated as the link $n_{a}+1$. If there is no contact between the end-effector and the external environment, the external force and torque at the end-effector are set to zero, i.e., fna+1ana+1=0 and nna+1ana+1=0.

**Figure 4:**
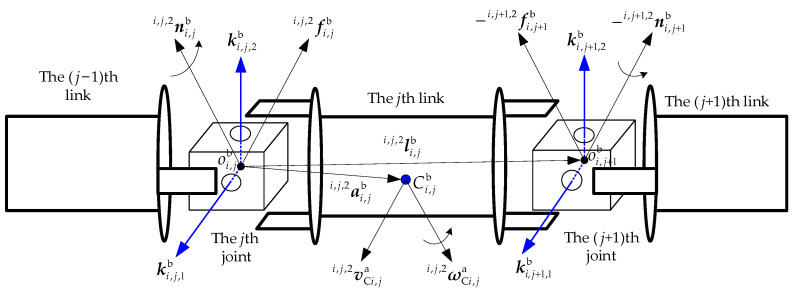
The force analysis for the *i*th link of Arm-b.

The recursive process for calculating the applied forces and torques on each joint of Arm-b is given as follows. The dynamic relationship of the *j*th link in the *i*th segment is shown in Figure 4.

The force and torque balance equations of the *j*th link in the *i*th segment are obtained as follows:

(14) fi,jbi,j,2=fi,j+1bi,j+1,2+mi,jb·v˙Ci,jbi,j,2ni,jbi,j,2=ni,j+1bi,j+1,2−fi,jbi,j,2×ai,jbi,j,2+fi,j+1bi,j+1,2×ai,jbi,j,2−li,jbi,j,2            +Ii,jbi,j,2·ω˙Ci,jbi,j,2+ωCi,jbi,j,2×Ii,jbi,j,2·ωCi,jbi,j,2

where fi,jbi,j,2 and ni,jbi,j,2 denote the force and torque exerted by the (*j* − 1)th link on the *j*th link. fi+1ai and ni+1ai are the force and torque exerted by the *j*th link on the (*j* + 1)th link. li,jbi,j,2 is the position vector from the *j*th joint to the (*j* + 1)th joint. They are all expressed in the frame attached to the second rotation axis of the *j*th joint.

Substituting Equation (9) into Equation (14), Equation (14) can be rewritten as follows.

(15) fi,jbi,j,2=fi,j+1bi,j+1,2+mi,jb·v˙i,jbi,j,2+ω˙i,jbi,j,2×ai,jbi,j,2+ωi,jbi,j,2×ωi,jbi,j,2×ai,jbi,j,2ni,jbi,j,2=ni,j+1bi,j+1,2−fi,jbi,j,2×ai,jbi,j,2+fi,j+1bi,j+1,2×ai,jbi,j,2−li,jbi,j,2               +Ii,jbi,j,2·ω˙i,jbi,j,2+ωi,jbi,j,2×Ii,jbi,j,2·ωi,jbi,j,2

Similarly, a virtual link appears in the above iteration, which does not exist for the actual structure of Arm-b. The treatment for this case is consistent with that of Arm-a.

## 4. Pose Stabilization of the Base by the PD Control with Feedforward Compensation

### 4.1. The Strategy for Pose Stabilization During Transposition

**Figure 5:**
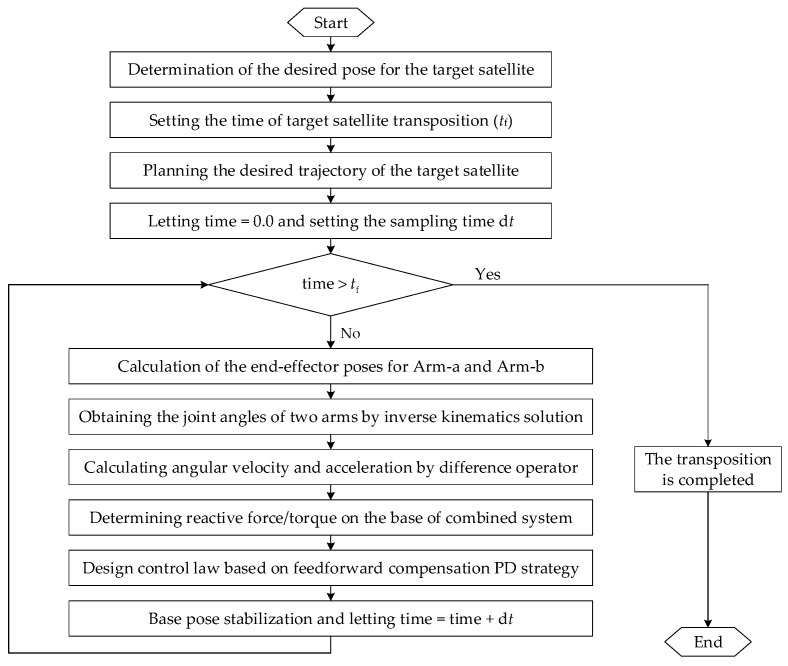
The flowchart of pose stabilization for the base of the combined system.

During target satellite transposition, the pose stabilization strategy for the base of the combined system is depicted in Figure 5. In this process, the non-offset discrete arm (Arm-a) is the mission manipulator to move the target satellite from its initial pose to its desired pose. At the same time, the segmented cable-driven hyper-redundant serpentine arm (Arm-b) serves as the auxiliary manipulator for monitoring the operation of Arm-a. Firstly, the desired pose of the target satellite was determined and the time (i.e., *t*_f_) was set based on transposition requirements. Accordingly, the target’s movement trajectory is planned using an interpolation method with a sampling time d*t*. After that, the pose of the target satellite is known at each moment.

Then, the system checked whether the current time exceeds the allotted mission time. If it is true, it means that the target satellite has been moved to the desired pose. Otherwise, the expected poses of end-effectors are calculated according to the geometric constraints between the target satellite and the two arms. Specifically, the relative poses between the target satellite’s center of mass, the capture point (Arm-a’s end-effector), and the monitoring point (Arm-b’s end-effector) remains fixed during transposition. Joint angular velocities and accelerations are determined by the inverse kinematics solution. The angular velocity and acceleration are obtained by the difference method. Furthermore, the PD control law integrated with dynamic feedforward compensation is designed to maintain the base stability. The feedforward compensation term is used to compensate for the base reaction force and torque generated by the motion of Arm-a and Arm-b, which can be calculated by the Newton–Euler recursion discussed in Section 3. The proportional-derivative control component is obtained according to the pose deviation of the base. Finally, the desired control force and torque are achieved by thrusters and reaction wheels installed on the base. This ensures the base remains stable while the DSHDASR performed the cooperative transposition-and-monitoring task.

### 4.2. Trajectory Planning of the Target Satellite

**Figure 6:**
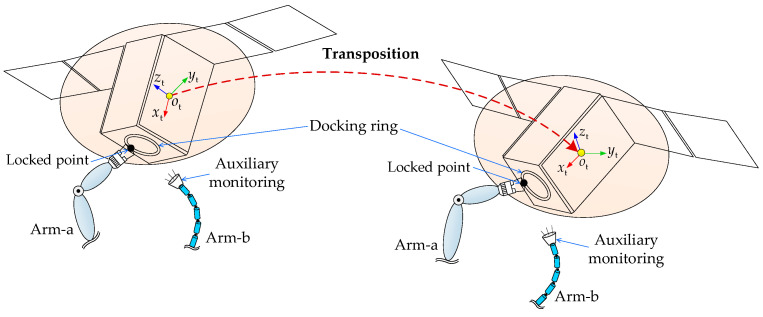
Target satellite transposition from the initial pose to the desired pose.

The schematic diagram for the target satellite transposition process is given in Figure 6. Before the transposition, the target satellite has been captured by Arm-a and the locked point is the capture point that is on the docking ring. During transposition, a monitoring device on Arm-b’s end-effector provided auxiliary monitoring of Arm-a’s operation. Therefore, the poses of Arm-a tip and Arm-b tip are dependent on the pose of the target satellite’s center of mass. Since the transposition is to move the target satellite from the initial pose to the desired pose, it can be taken as point-to-point motion planning. To ensure smooth start and stop transitions, the trajectory for the target satellite’s center of mass was generated using quintic polynomial interpolation.

The initial pose and the desired pose of the target are set as follows:

(16) $$\left\{\begin{array}{l} X_{t_{0}}={\left[\begin{array}{cccccc} p_{t_{0}\_x}, & p_{t_{0}\_y}, & p_{t_{0}\_z}, & \alpha_{t_{0}\_z}, & \alpha_{t_{0}\_y}, & \alpha_{t_{0}\_x} \end{array}\right]}^{T}\\ X_{t_{f}}={\left[\begin{array}{cccccc} p_{t_{f}\_x}, & p_{t_{f}\_y}, & p_{t_{f}\_z}, & \alpha_{t_{f}\_z}, & \alpha_{t_{f}\_y}, & \alpha_{t_{f}\_x} \end{array}\right]}^{T} \end{array}\right.$$

where $p_{t_{0}\_x}$, $p_{t_{0}\_y}$ and $p_{t_{0}\_z}$ are initial positions of the target satellite’s centroid along *x*-axis, *y*-axis, and *z*-axis, respectively. The desired positions are $p_{t_{f}\_x}$, $p_{t_{f}\_y}$, and $p_{t_{f}\_z}$. The initial attitude angles (Z-Y-X Euler angles) are denoted by $\alpha_{t_{0}\_z}$, $\alpha_{t_{0}\_y}$, and $\alpha_{t_{0}\_x}$. Their desired attitude angles are $\alpha_{t_{f}\_z}$, $\alpha_{t_{f}\_y}$, and $\alpha_{t_{f}\_x}$, respectively. The velocity and acceleration are set to zeros at the initial and terminal states. Therefore, the desired motion trajectory of the target satellite is obtained as follows.

(17) $$X_{\mathrm{td}}\left(t\right)=X_{t_{0}}+\frac{10\left(X_{t_{f}}-X_{t_{0}}\right)}{{t_{f}}^{3}}t^{3}-\frac{15\left(X_{t_{f}}-X_{t_{0}}\right)}{{t_{f}}^{4}}t^{4}+\frac{6\left(X_{t_{f}}-X_{t_{0}}\right)}{{t_{f}}^{5}}t^{5},0\leq t\leq t_{f}$$

According to the pose relationship among the target satellite’s centroid, Arm-a tip and Arm-b tip, the desired trajectories of the two arms are also calculated. Specifically, the desired poses of Arm-a tip and Arm-b tip are obtained, i.e., $P_{\mathrm{ed}}^{a}$, $R_{\mathrm{ed}}^{a}$, $P_{\mathrm{ed}}^{b}$, and $R_{\mathrm{ed}}^{b}$.

### 4.3. Feedforward Compensation PD Control Algorithm

**Figure 7:**
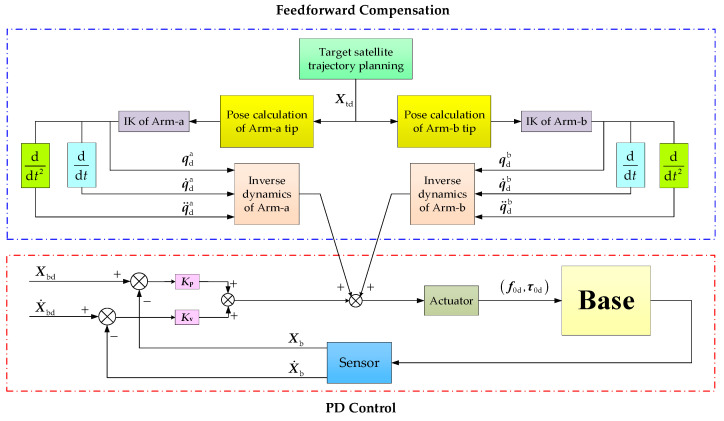
The flow chart of the PD control with feedforward compensation.

As shown in Figure 7, the control method for the base’s pose stabilization is composed of two components. The former is dynamic feedforward compensation, while the latter is proportional-derivative control. The detailed calculation process for each component is given as follows.

#### 4.3.1. Calculation of Feedforward Compensation

As discussed above, once the motion trajectory for the target satellite’s centroid was planned, the required end-effector poses for Arm-a and Arm-b became known. The joint angles, velocities, and accelerations of Arm-a and Arm-b are obtained by the inverse kinematics solution and numerical differentiation. These joint motion parameters are then fed into the Newton–Euler recursive algorithm to compute the corresponding joint forces and torques. Notably, the last link of Arm-a and the target satellite form a single rigid body after the target is captured. Therefore, the actual mass properties of the link used in the Newton–Euler recursion are changed, and they can be obtained as follows.

**Figure 8:**
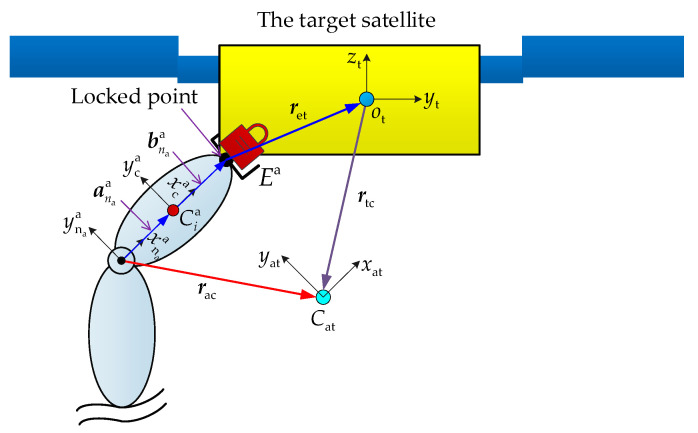
The equivalent last link of Arm-a constituted by the link and the target satellite.

As shown in Figure 8, {$x_{n_{a}}^{a}$, $y_{n_{a}}^{a}$, $z_{n_{a}}^{a}$} is the frame fixed on the last joint of Arm-a. {$x_{t}$, $y_{t}$, $z_{t}$} is the frame attached to the target satellite. The equivalent centroid of the last link is denoted by $C_{\mathrm{at}}$. Its associated frame is {$x_{\mathrm{at}}$, $y_{\mathrm{at}}$, $z_{\mathrm{at}}$}. The position vector of $C_{\mathrm{at}}$ expressed in {$x_{n_{a}}^{a}$, $y_{n_{a}}^{a}$, $z_{n_{a}}^{a}$} is obtained as follows:

(18) $$r_{\mathrm{ac}}=\frac{m_{n_{a}}^{a}a_{n_{a}}^{a}+m_{t}\left(a_{n_{a}}^{a}+b_{n_{a}}^{a}+r_{\mathrm{et}}\right)}{m_{n_{a}}^{a}+m_{t}}$$

where $m_{n_{a}}^{a}$ and $m_{t}$ are the masses of the last link and the target, respectively. $r_{\mathrm{et}}$ is the position vector from Arm-a tip to the centroid of the target satellite.

The equivalent inertia tensor expressed in the {$x_{n_{a}}^{a}$, $y_{n_{a}}^{a}$, $z_{n_{a}}^{a}$} is obtained as follows:

(19) Iac=Inaa+mnaaracT·racE−rac·racT+RtnaItRtTna+mtrtcT·rtcE−rtc·rtcT

where $I_{n_{a}}^{a}$ and $I_{t}$ are the inertia tensors of the last link and the target, respectively, expressed in their attached coordinate systems. E is the unit matrix. Rtna is the rotation matrix from frame {$x_{t}$, $y_{t}$, $z_{t}$} to frame {$x_{n_{a}}^{a}$, $y_{n_{a}}^{a}$, $z_{n_{a}}^{a}$}. $r_{\mathrm{tc}}$ is the position vector from the centroid of the target satellite to $C_{\mathrm{at}}$.

Replace $m_{n_{a}}^{a}$, $a_{n_{a}}^{a}$, and $I_{n_{a}}^{a}$ with $m_{n_{a}}^{a}+m_{t}$, $r_{\mathrm{ac}}$, and $I_{\mathrm{ac}}$. These equivalent parameters, along with the joint motion data, are then substituted into (13) and (15) to yield the joint forces and torques. Subsequently, reaction forces (−f1a1 and −f1,1b1,1,2) and torques (−n1a1 and −n1,1b1,1,2) on the base induced by the two arms are obtained. By expressing these forces and torques in the inertial frame, the dynamic feedforward compensation term is derived as follows:

(20) fbDFC=−R1a·f1a1+R1,1,2b·f1,1b1,1,2nbDFC=−R1a·n1a1+b0a×R1a·f1a1+R1,1,2b·n1,1b1,1,2+b0b×R1,1,2b·f1,1b1,1,2

where $R_{1}^{a}$ is the rotation matrix from the frame fixed on the first joint of Arm-a to the inertial frame. $R_{1,1,2}^{b}$ refers to the rotation matrix from the frame attached to the second rotational axis of the first joint of Arm-b to the inertial frame.

#### 4.3.2. Control Law Design

As shown in Figure 7, the pose stability control of the base consists of the dynamic feedforward compensation term and the PD control term. The former is calculated using Equation (20). The PD control term is derived from the deviation between the base’s desired and measured poses. In practice, the desired pose is the base’s state when the target satellite is grasped. The base’s current pose is measured by onboard sensors (e.g., gyroscopes and accelerometers). Therefore, the PD control term is derived as follows:

(21) $$\left\{\begin{array}{l} f_{b}^{\mathrm{PD}}=-K_{\mathrm{pp}}\cdot \Delta X_{p}-K_{\mathrm{vv}}\cdot \Delta X_{v}\\ n_{b}^{\mathrm{PD}}=-K_{p\varphi }\cdot \Delta X_{\varphi }-K_{v\omega }\cdot \Delta X_{\omega } \end{array}\right.$$

where $\Delta X_{p}$ and $\Delta X_{\varphi }$ are the position and attitude deviations for the base. Their linear and angular deviations are denoted by $\Delta X_{v}$ and $\Delta X_{\omega }$. $K_{\mathrm{pp}}$ and $K_{p\varphi }$ are the proportional gain matrices corresponding to the position deviation and attitude deviation, respectively. $K_{\mathrm{vv}}$ and $K_{v\omega }$ are the derivative gain matrices.

In this paper, the PD gains are designed following a systematic performance and robustness trade-off principle. Firstly, the gains should be proportional to the mass/inertia of the base, ensuring the physical consistency of the control force/torque. Furthermore, the gain matrix should be positive and definite to ensure system stability. Finally, to ensure system robustness, the minimum eigenvalue of the proportional gain matrix should be set in consideration of the magnitude of external disturbances and the required control accuracy. In other words, the proportional gain is expressed in a form explicitly related to the base mass, i.e., $K_{\mathrm{pp}}=m_{0}{\tilde{K}}_{\mathrm{pp}}$. Here, $m_{0}$ denotes the base mass, and ${\tilde{K}}_{\mathrm{pp}}$ represents a mass-independent normalized proportional gain that characterizes the desired closed-loop dynamic behavior.

Combining Equations (20) and (21), the control law of the base’s pose stabilization is obtained as follows.

(22) fbd=fbDFC+fbPD=−KppΔXp−KvvΔXv−R1af1a1+R1,1,2bf1,1b1,1,2nbd=nbDFC+nbPD       =−KpφΔXφ−KvωΔXω−R1an1a1+b0a×R1af1a1+R1,1,2bn1,1b1,1,2+b0b×R1,1,2bf1,1b1,1,2

#### 4.3.3. Generation of Control Force and Torque

**Figure 9:**
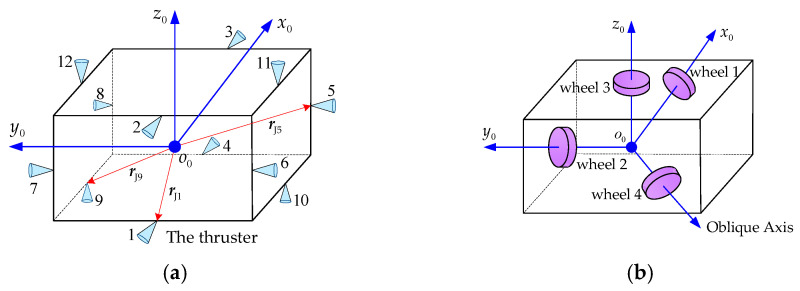
The configuration of thrusters and reaction wheels on the base. (**a**) The layout of thrusters. (**b**) The layout of reaction wheels.

The control forces and torques calculated from Equation (22) are generated by thrusters and reaction wheels. As shown in Figure 9, the layout of the thrusters (see Figure 9a) and reaction wheels (see Figure 9b) installed on the base is illustrated. According to Figure 9a, twelve thrusters are mounted on the base, organized into three groups aligned with the *x*_0_, *y*_0_, and *z*_0_ axes of the base-fixed frame. Group 1 includes four thrusters that are numbered 1, 2, 3, and 4, respectively. These thrusters are arranged on the *x*_0_-*z*_0_ plane. Thrusters 1 and 2 produce thrust along the positive *x*_0_ axis direction, while Thrusters 3 and 4 generate thrust along the negative *x*_0_ axis direction. Group 2 consists of Thrusters 5, 6, 7, and 8, which are placed on the *x*_0_-*y*_0_ plane and Group 3 includes Thrusters 9, 10, 11, and 12, which are arranged on the *y*_0_-*z*_0_ plane. The definition of the thrust direction for these thrusters in Group 2 and Group 3 is the same as that of the thrusters in Group 1.

The position vector of the *i*th thruster in the frame fixed on the base centroid is denoted as $r_{Ji}$. To generate a desired control force along a principal axis, two opposing thrusters from the corresponding group should be fired. For example, Thrusters 1 and 4 are selected to generate the desired control force along the *x*_0_ axis; Thrusters 5 and 8 are used for *y*_0_ axis; and Thrusters 9 and 12 are selected for the *z*_0_ axis. Thus, the desired control force from Equation (22) can be implemented as follows:

(23) $$\left\{\begin{array}{lll} f_{J1}=f_{\mathrm{bd}}(1), & f_{J4}=0, & if\;\;f_{\mathrm{bd}}(1)>0\\ f_{J1}=0, & f_{J4}=f_{\mathrm{bd}}(1), & if\;f_{\mathrm{bd}}(1)<0\\ f_{J1}=0, & f_{J4}=0, & if\;f_{\mathrm{bd}}(1)=0 \end{array}\right.$$

(24) $$\left\{\begin{array}{lll} f_{J5}=f_{\mathrm{bd}}(2), & f_{J8}=0, & if\;f_{\mathrm{bd}}(2)>0\\ f_{J5}=0, & f_{J8}=f_{\mathrm{bd}}(2), & if\;f_{\mathrm{bd}}(2)<0\\ f_{J5}=0, & f_{J8}=0, & if\;f_{\mathrm{bd}}(2)=0 \end{array}\right.$$

(25) $$\left\{\begin{array}{lll} f_{J9}=f_{\mathrm{bd}}(3), & f_{J12}=0, & if\;f_{\mathrm{bd}}(3)>0\\ f_{J9}=0, & f_{J12}=f_{\mathrm{bd}}(3), & if\;f_{\mathrm{bd}}(3)<0\\ f_{J9}=0, & f_{J12}=0, & if\;f_{\mathrm{bd}}(3)=0 \end{array}\right.$$

where $f_{Ji}$ is the thrust generated by the *i*th thruster. $f_{\mathrm{bd}}(1)$, $f_{\mathrm{bd}}(2)$, and $f_{\mathrm{bd}}(3)$ are the components of the vector $f_{\mathrm{bd}}$ along the *x*_0_, *y*_0_, and *z*_0_ axes, respectively.

The layout of the reaction wheels is depicted in Figure 9b. Wheels 1, 2, and 3 are installed along the *x*_0_, *y*_0_, and *z*_0_ axes, respectively. Each wheel generates the control torque to adjust the attitude about its corresponding axis. Wheel 4 is oriented along an oblique axis to serve as a backup. In case of a failure in any one of Wheels 1–3, it is activated to maintain base attitude control.

Once the desired control torque in the feedforward compensation PD control algorithm is determined, it can be generated through rotation of the reaction wheels. Since the thrust direction of each thruster does not pass through the center of mass of the base, their operation introduces disturbance torques to the base. Therefore, the total torque output from the reaction wheels has two components, namely the nominal control torque from the algorithm and a compensatory torque to cancel thruster-induced disturbances. Taking Thrusters 1, 4, 5, 8, 9, and 12 as an example, the disturbance torque generated by their thrusts is obtained as follows.

(26) $$\begin{array}{ll} n_{\mathrm{ext}} & =r_{J1}\times {\left[\begin{array}{ccc} f_{J1} & 0 & 0 \end{array}\right]}^{T}+r_{J4}\times {\left[\begin{array}{ccc} -f_{J4} & 0 & 0 \end{array}\right]}^{T}\\ & +r_{J5}\times {\left[\begin{array}{ccc} 0 & f_{J5} & 0 \end{array}\right]}^{T}+r_{J8}\times {\left[\begin{array}{ccc} 0 & -f_{J8} & 0 \end{array}\right]}^{T}\\ & +r_{J9}\times {\left[\begin{array}{ccc} 0 & 0 & f_{J9} \end{array}\right]}^{T}+r_{J12}\times {\left[\begin{array}{ccc} 0 & 0 & -f_{J12} \end{array}\right]}^{T} \end{array}$$

Combining Equations (22) and (26) yields the total three-axis control torque required from the reaction wheels.

(27) $$n_{\mathrm{rw}}=n_{\mathrm{bd}}+n_{\mathrm{ext}}$$

Assuming that Reaction Wheels 1, 2, and 3 are operating nominally, the relationship between the control torque and its angular acceleration is given as follows:

(28) $$n_{\mathrm{rw}}=\left[\begin{array}{ccc} I_{\mathrm{rwx}} & 0 & 0\\ 0 & I_{\mathrm{rwy}} & 0\\ 0 & 0 & I_{\mathrm{rwz}} \end{array}\right]\left[\begin{array}{c} {\dot{\omega }}_{\mathrm{rwx}}\\ {\dot{\omega }}_{\mathrm{rwy}}\\ {\dot{\omega }}_{\mathrm{rwz}} \end{array}\right]$$

where $I_{\mathrm{fwx}}$, $I_{\mathrm{rwy}}$, and $I_{\mathrm{rwz}}$ stand for moments of inertia of Reaction Wheels 1, 2, and 3, respectively. ${\dot{\omega }}_{\mathrm{rwx}}$, ${\dot{\omega }}_{\mathrm{rwy}}$, and ${\dot{\omega }}_{\mathrm{rwz}}$ refer to angular accelerations of Reaction Wheels 1, 2, and 3, respectively.

Substituting Equation (27) into Equation (28), the desired angular acceleration of each reaction wheel is calculated as follows.

(29) $$\left[\begin{array}{c} {\dot{\omega }}_{\mathrm{rwx}}\\ {\dot{\omega }}_{\mathrm{rwy}}\\ {\dot{\omega }}_{\mathrm{rwz}} \end{array}\right]={\left[\begin{array}{ccc} I_{\mathrm{rwx}} & 0 & 0\\ 0 & I_{\mathrm{rwy}} & 0\\ 0 & 0 & I_{\mathrm{rwz}} \end{array}\right]}^{-1}\left(n_{\mathrm{bd}}+n_{\mathrm{ext}}\right)$$

Finally, the pose stabilization control of the combined system’s base is achieved by operating thrusters and reaction wheels according to Equations (23)–(25) and (29).

It is worth noting that actuator saturation can disrupt the controller’s linearity and may impair stability. To address this issue, the saturation limits are discussed as follows. From the perspective of control force and torque, their magnitude are determined by the feedforward compensation and the PD control term. Among these, the feedforward compensation contributes the majority, while the PD term accounts for a smaller portion. Therefore, to ensure that the feedforward compensation does not induce actuator saturation, a limiter has been applied to it. The specific settings are given as follows:

(30) $$f_{b}^{\mathrm{PD}}=\left\{\begin{array}{ll} \left(f_{b,\max}^{\mathrm{PD}}/\left\|f_{b}^{\mathrm{PD}}\right\|\right)\cdot \left\|f_{b}^{\mathrm{PD}}\right\|, & \mathrm{if}\;\left\|f_{b}^{\mathrm{PD}}\right\|>f_{b,\max}^{\mathrm{PD}}\\ f_{b}^{\mathrm{PD}}, & \mathrm{else} \end{array}\right.$$

(31) $$n_{b}^{\mathrm{PD}}=\left\{\begin{array}{ll} \left(n_{b,\max}^{\mathrm{PD}}/\left\|n_{b}^{\mathrm{PD}}\right\|\right)\cdot n_{b}^{\mathrm{PD}}, & \mathrm{if}\;\left\|n_{b}^{\mathrm{PD}}\right\|>n_{b,\max}^{\mathrm{PD}}\\ n_{b}^{\mathrm{PD}}, & \mathrm{else} \end{array}\right.$$

where $f_{b,\max}^{\mathrm{PD}}$ and $n_{b,\max}^{\mathrm{PD}}$ are the maximum output force and torque of the actuator.

### 4.4. Analysis of the Proposed Algorithm

#### 4.4.1. Stability Analysis

The movement of the base is composed of the translation and rotation. According to Newton–Euler formulation, the dynamics model of the base can be obtained as follows:

(32) $$m_{0}{\dot{v}}_{0}=f_{\mathrm{bd}}+f_{\mathrm{arm}}+f_{\mathrm{ext}}$$

(33) $$I_{0}{\dot{\omega }}_{0}+\omega_{0}\times I_{0}\omega_{0}=n_{\mathrm{bd}}+n_{\mathrm{arm}}+n_{\mathrm{ext}}$$

where $m_{0}$ and $I_{0}$ are the mass and inertia of the base. Its linear velocity and angular velocity are $v_{0}$ and $\omega_{0}$, respectively. $f_{\mathrm{arm}}$ and $n_{\mathrm{arm}}$ are the reaction force and reaction torque on the base induced by the two arms. $f_{\mathrm{ext}}$ and $n_{\mathrm{ext}}$ are the external disturbance force and torque, respectively.

Based on Equation (22), the force and torque of PD control with feedforward compensation are obtained. Substituting Equation (22) into Equations (32) and (33), it is calculated as follows:

(34) $$m_{0}{\dot{v}}_{0}=-K_{\mathrm{pp}}\Delta X_{p}-K_{\mathrm{vv}}\Delta X_{v}+f_{b}^{\mathrm{DFC}}+f_{\mathrm{arm}}+f_{\mathrm{ext}}$$

(35) $$I_{0}{\dot{\omega }}_{0}+\omega_{0}\times I_{0}\omega_{0}=-K_{p\varphi }\Delta X_{\varphi }-K_{v\omega }\Delta X_{\omega }+n_{b}^{\mathrm{DFC}}+n_{\mathrm{arm}}+n_{\mathrm{ext}}$$

where $\Delta X_{p}=p_{0}-p_{0d}$, $\Delta X_{v}={\dot{p}}_{0}-{\dot{p}}_{0d}={\dot{p}}_{0}$. Because the base undergoes only small variations in its attitude, $\Delta X_{\varphi }$ and $\Delta X_{\omega }$ can be calculated by $\Delta X_{\varphi }=\varphi_{0}-\varphi_{0d}$ and $\Delta X_{\omega }={\dot{\varphi }}_{0}-{\dot{\varphi }}_{0d}={\dot{\varphi }}_{0}$. $p_{0}$ and $\varphi_{0}$ are the current position and attitude of the base. $p_{0d}$ and $\varphi_{0d}$ are the desired position and attitude. Thus, ${\dot{p}}_{0}$ and ${\dot{\varphi }}_{0}$ are the current linear velocity and angular velocity of the base. ${\dot{p}}_{0d}$ and ${\dot{\varphi }}_{0d}$ are the desired linear velocity and angular velocity, and they are set as zeros in this paper. Therefore, $v_{0}={\dot{p}}_{0}$, ${\dot{v}}_{0}={\ddot{p}}_{0}$, $\omega_{0}={\dot{\varphi }}_{0}$, and ${\dot{\omega }}_{0}={\ddot{\varphi }}_{0}$ can be obtained.

From a reformulation of Equations (34) and (35), the closed-loop dynamics for the base’s position and attitude can be derived as follows:

(36) $$m_{0}{\ddot{p}}_{0}+K_{\mathrm{pp}}\Delta X_{p}+K_{\mathrm{vv}}{\dot{p}}_{0}=\delta_{f}$$

(37) $$I_{0}{\ddot{\varphi }}_{0}+K_{p\varphi }\Delta X_{\varphi }+K_{v\omega }{\dot{\varphi }}_{0}=\delta_{\tau }$$

where $\delta_{f}=f_{b}^{\mathrm{DFC}}+f_{\mathrm{arm}}+f_{\mathrm{ext}}$ and $\delta_{\tau }=n_{b}^{\mathrm{DFC}}+n_{\mathrm{arm}}+n_{\mathrm{ext}}-\omega_{0}\times I_{0}\omega_{0}$. $\delta_{f}$ and $\delta_{\tau }$ are defined as the lumped disturbance in the position channel and in attitude channel, respectively.

Assuming that $\delta_{f}$ and $\delta_{\tau }$ are zeros, Equations (36) and (37) can be rewritten as follows.

(38) $${\ddot{p}}_{0}=\left(-K_{\mathrm{pp}}\Delta X_{p}-K_{\mathrm{vv}}{\dot{p}}_{0}\right)/m_{0}$$

(39) $${\ddot{\varphi }}_{0}={I_{0}}^{-1}\left(-K_{p\varphi }\Delta X_{\varphi }-K_{v\omega }{\dot{\varphi }}_{0}\right)$$

The Lyapunov function of the control system is established as follows.

(40) $$\begin{array}{ll} V & =V_{p}+V_{\varphi }=\frac{1}{2}\left(\Delta {X_{v}}^{T}m_{0}\Delta X_{v}+\Delta {X_{p}}^{T}K_{\mathrm{pp}}\Delta X_{p}\right)+\frac{1}{2}\left(\Delta {X_{\omega }}^{T}I_{0}\Delta X_{\omega }+\Delta {X_{\varphi }}^{T}K_{p\varphi }\Delta X_{\varphi }\right)\\ & =\frac{1}{2}\left({{\dot{p}}_{0}}^{T}m_{0}{\dot{p}}_{0}+\Delta {X_{p}}^{T}K_{\mathrm{pp}}\Delta X_{p}\right)+\frac{1}{2}\left({{\dot{\varphi }}_{0}}^{T}I_{0}{\dot{\varphi }}_{0}+\Delta {X_{\varphi }}^{T}K_{p\varphi }\Delta X_{\varphi }\right) \end{array}$$

Owing to the fact that $m_{0}$, $K_{\mathrm{pp}}$, $I_{0}$, and $K_{p\varphi }$ are all positive definite matrix values, V is greater than or equal to zero. Furthermore, only when ${\dot{p}}_{0}$, $\Delta X_{p}$, ${\dot{\varphi }}_{0}$, and $\Delta X_{\varphi }$ are zeros, V is equal to zero. Differentiating Equation (40), it can be obtained as follows.

(41) $$\dot{V}={{\dot{p}}_{0}}^{T}m_{0}{\ddot{p}}_{0}+\Delta {X_{p}}^{T}K_{\mathrm{pp}}{\dot{p}}_{0}+{{\dot{\varphi }}_{0}}^{T}I_{0}{\ddot{\varphi }}_{0}+\Delta {X_{\varphi }}^{T}K_{p\varphi }{\dot{\varphi }}_{0}$$

Substituting Equations (38) and (39) into Equation (41), we can obtain

(42) $$\dot{V}=-{{\dot{p}}_{0}}^{T}K_{\mathrm{vv}}{\dot{p}}_{0}-{{\dot{\varphi }}_{0}}^{T}K_{v\omega }{\dot{\varphi }}_{0}\leq 0$$

According to Equation (42), the control system is globally asymptotically stable.

The stability analysis presented above is derived under nominal conditions, where the dynamic feedforward compensation is assumed to exactly cancel the base coupling disturbances induced by the motion of the manipulators and the captured target satellite. Under this ideal assumption, the closed-loop system errors are guaranteed to asymptotically converge to zero.

In practical implementations, however, the perfect feedforward cancellation of coupling effects is difficult to achieve due to modeling uncertainties, parameter deviations, and unmodeled dynamics. In this case, the system is subject to bounded residual disturbances. Based on Lyapunov stability theory, it can be shown that the closed-loop system remains uniformly ultimately bounded, where the tracking errors converge to a bounded neighborhood whose size depends on the upper bound of the disturbances, rather than strictly converging to zero.

#### 4.4.2. Robustness Analysis

Based on Equations (36) and (37), the lumped disturbances in position and attitude channels are $\delta_{f}$ and $\delta_{\tau }$. They can both be decomposed into two components. The former is the feedforward compensation error, which could be generated due to parameter uncertainties or modeling errors. The latter is the disturbance force (or torque) exerted by the environment. When the base reaches the stable state, ${\ddot{p}}_{0}$, ${\dot{p}}_{0}$, ${\dot{\varphi }}_{0}$, and ${\ddot{\varphi }}_{0}$ are all zeros. Therefore, its position and attitude errors can be obtained as follows.

(43) $$\Delta X_{p0}={\left(K_{\mathrm{pp}}\right)}^{-1}\cdot \delta_{f},\;\;\;\;\Delta X_{\varphi }={\left(K_{p\varphi }\right)}^{-1}\cdot \delta_{\tau }$$

Error bounds for the pose of the base are determined as follows:

(44) $$\left\{\begin{array}{l} e_{p}\leq \left\|{\left(K_{\mathrm{pp}}\right)}^{-1}\right\|\left\|\delta_{f}\right\|=\frac{1}{\lambda_{\min}\left(K_{\mathrm{pp}}\right)}\left\|\delta_{f}\right\|\\ e_{a}\leq \left\|{\left(K_{p\varphi }\right)}^{-1}\right\|\left\|\delta_{\tau }\right\|=\frac{1}{\lambda_{\min}\left(K_{p\varphi }\right)}\left\|\delta_{\tau }\right\| \end{array}\right.$$

where $\lambda_{\min}(\cdot )$ is the minimum eigenvalue of a matrix.

According to Equation (44), the position and attitude errors are related to external disturbances and proportional gain. Additionally, the larger minimum eigenvalue of the proportional gain results in a smaller error. As a result, we can enhance the robustness of the system by increasing the minimum eigenvalue of the proportional gain matrix.

#### 4.4.3. Gain Margins Analysis

The system can be decoupled into six independent second-order systems.

(45) $$\left\{\begin{array}{l} m_{0}{\ddot{p}}_{0}(i)+K_{\mathrm{pp}}(i,i)\Delta X_{p}(i)+K_{\mathrm{vv}}(i,i){\dot{p}}_{0}(i)=\delta_{f}(i)\\ I_{0}(i,i){\ddot{\varphi }}_{0}(i)+K_{p\varphi }(i,i)\Delta X_{\varphi }(i)+K_{v\omega }(i,i){\dot{\varphi }}_{0}(i)=\delta_{\tau }(i) \end{array}\right.\;\;\;i=1,2,3$$

For the position channel, the open-loop transfer function for each axis is given as follows.

(46) $$G_{i}^{p}\left(s\right)=\frac{K_{\mathrm{vv}}(i,i)s+K_{\mathrm{pp}}(i,i)}{ms^{3}}e^{-\tau s}$$

The phase crossover frequency $\omega_{i}^{\mathrm{pc}}$ satisfies the following equation.

(47) $$\arctan\left(\frac{K_{\mathrm{vv}}(i,i)\omega_{i}^{\mathrm{pc}}}{K_{\mathrm{pp}}(i,i)}\right)-\frac{\pi }{2}-\tau \omega_{i}^{\mathrm{pc}}=-\pi$$

As derived from Equation (47), $\omega_{i}^{\mathrm{pc}}$ can be determined by using numerical methods such as the Newton–Raphson method or the bisection method. The corresponding gain at this frequency are calculated as follows.

(48) $$\left|L_{i}^{p}\left(j\omega_{i}^{\mathrm{pc}}\right)\right|=\frac{\sqrt{{\left[K_{\mathrm{pp}}(i,i)\right]}^{2}+{\left[K_{\mathrm{vv}}(i,i)\omega_{i}^{\mathrm{pc}}\right]}^{2}}}{m_{0}{\left(\omega_{i}^{\mathrm{pc}}\right)}^{2}}\cdot \frac{1}{\sqrt{1+{\left(\tau \omega_{i}^{\mathrm{pc}}\right)}^{2}}}$$

Thus, the gain margin of the position channel is obtained as follows.

(49) $$G_{M,i}^{p}=\frac{1}{\left|L_{i}^{p}\left(j\omega_{i}^{\mathrm{pc}}\right)\right|},\;\;\;G_{M,\mathrm{dB},i}^{p}=-20\mathrm{lg}\left(\left|L_{i}^{p}\left(j\omega_{i}^{\mathrm{pc}}\right)\right|\right)$$

Similarly, the gain margin for the attitude channel can also be calculated, which is omitted here for brevity. It should be noted that the gain margin analysis presented in this section is conducted around the nominal operating point based on a linearized model. As such, it serves primarily as a theoretical stability reference and has inherent limitations when applied to the nonlinear and time-varying dynamics of the base–manipulator–target combined system, particularly under large payload conditions.

#### 4.4.4. Sensitivity Analysis

During the transposition, the desired velocity and acceleration of the base are zeros, i.e., ${\dot{p}}_{0d}=0$ and ${\dot{\varphi }}_{0d}=0$. Substituting them into Equation (45), the closed-loop control system for the base is established as follows.

(50) $$\left\{\begin{array}{l} m_{0}\Delta {\ddot{X}}_{p}(i)+K_{\mathrm{vv}}(i,i)\Delta {\dot{X}}_{p}(i)+K_{\mathrm{pp}}(i,i)\Delta X_{p}(i)=\delta_{f}(i)\\ I_{0}(i,i)\Delta {\ddot{X}}_{\varphi }(i)+K_{v\omega }(i,i)\Delta {\dot{X}}_{\varphi }(i)+K_{p\varphi }(i,i)\Delta X_{\varphi }(i)=\delta_{\tau }(i) \end{array}\right.\;\;\;i=1,2,3$$

Based on Equation (50), the position or attitude channels can be treated as a second-order system that is fully defined by two characteristic parameters (damping ratio and natural frequency). Since the inherent parameters of a second-order system define its intrinsic dynamic characteristics, which remain unchanged by external disturbances, the disturbance force and torque can be neglected in Equation (50).

Taking the sensitivity analysis of parameter uncertainty as an example, assume that the true mass parameter of the base is $m_{0}=(1+\lambda_{m}){\tilde{m}}_{0}$. ${\tilde{m}}_{0}$ is the estimated mass of the base and $\lambda_{m}$ is the uncertainty. From Equation (50), the closed-loop characteristic equation for the position channel can be derived as

(51) $$s_{i}^{2}+\frac{K_{\mathrm{vv}}(i,i)}{\left(1+\lambda_{m}\right){\tilde{m}}_{0}}s_{i}+\frac{K_{\mathrm{pp}}(i,i)}{\left(1+\lambda_{m}\right){\tilde{m}}_{0}}=0\;\;\;i=1,2,3$$

From Equation (51), it can be seen that the uncertainty $\lambda_{m}$ affects the distribution of the system poles. When $m_{0}$ increases (i.e., $\lambda_{m}>0$), the poles move toward the imaginary axis, potentially making the response more oscillatory. In this case, the natural frequency decreases and the damping ratio reduces. When $m_{0}$ decreases (i.e., $\lambda_{m}<0$), the poles move away from the imaginary axis, leading to an increase in both the natural frequency and damping ratio. However, if $m_{0}$ becomes too small, the poles might approach the imaginary axis again and exacerbate oscillations.

The damping ratio and natural frequency can be calculated as follows.

(52) $$\zeta_{i}^{p}=\frac{K_{\mathrm{vv}}(i,i)}{2\sqrt{m_{0}K_{\mathrm{pp}}(i,i)}},\;\;\;\;\omega_{n,i}^{p}=\sqrt{\frac{K_{\mathrm{pp}}(i,i)}{m_{0}}}$$

For the position channel, the sensitivity of the damping ratio and natural frequency with respect to the mass parameter uncertainty are then obtained as follows.

(53) $$S_{\zeta }^{p}=\frac{\partial \zeta_{i}^{p}}{\partial m_{0}}\cdot \frac{m_{0}}{\zeta_{i}^{p}}=-\frac{1}{2},\;\;\;\;\;S_{\omega }^{p}=\frac{\partial \omega_{n,i}^{p}}{\partial m_{0}}\cdot \frac{m_{0}}{\omega_{n,i}^{p}}=-\frac{1}{2}$$

From Equation (53), it implies that a 10% increase in mass results in approximately a 5% decrease in both the damping ratio and natural frequency.

Similarly, the closed-loop characteristic function for the attitude channel can be established based on Equation (50). Its damping ratio and natural frequency can also be calculated. The corresponding sensitivity functions of the damping ratio and natural frequency with respect to the inertia parameter uncertainty are obtained as follows.

(54) $$S_{\zeta }^{\varphi }=\frac{\partial \zeta_{i}^{\varphi }}{\partial I_{0}(i,i)}\cdot \frac{I_{0}(i,i)}{\zeta_{i}^{\varphi }}=-\frac{1}{2},\;\;\;\;\;S_{\omega }^{\varphi }=\frac{\partial \omega_{n,i}^{\varphi }}{\partial I_{0}(i,i)}\cdot \frac{I_{0}(i,i)}{\omega_{n,i}^{\varphi }}=-\frac{1}{2}$$

where $\zeta_{i}^{\varphi }=K_{v\omega }(i,i)/\sqrt{4I_{0}(i,i)K_{p\varphi }(i,i)}$ and $\omega_{n,i}^{\varphi }=\sqrt{K_{p\varphi }(i,i)/I_{0}(i,i)}$. A 10% increase in inertia reduces both the natural frequency and damping ratio by about 5%. This trend is fully analogous to the position channel analysis.

#### 4.4.5. Discussion of the Challenges

Regarding the time delay issue, system delays from sensor sampling, communication, and computation could introduce phase errors, particularly degrading the performance of the model-dependent feedforward compensation. This would place a greater burden on the robustness of the PD feedback loop. We will study some strategies like predictive filtering or high-speed data protocols to address this issue.

On the problem of computational cost, the real-time execution of the recursive Newton–Euler algorithm for feedforward dynamics, while efficient (*o*(*n*)), must be rigorously validated on representative flight processor hardware.

About the actuator saturation, the physical limits of the thrusters and reaction wheels are the critical constraint. Each actuator requires integrating a saturation management block and a control allocation strategy to distribute torque among these actuators, preventing performance degradation or instability when commanded values exceed physical limits.

## 5. Simulation Study

**Figure 10:**
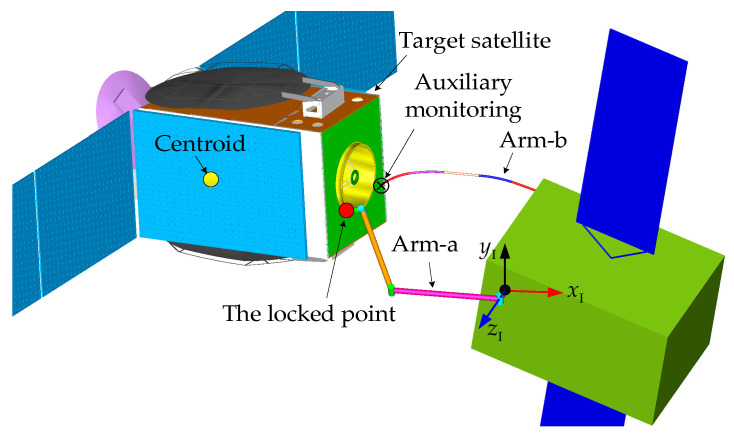
The initial state of the combined system in the simulation.

To verify the proposed method, a co-simulation model is built using Matlab2016 and Adams2018 software. As shown in Figure 10, the DSHDASR for cooperatively transposing the target satellite is taken as an example in the simulation. At the initial moment of the simulation, the target has been grasped by the DSHDASR. The capture point is on the docking ring of the target, and it is referred to as the locked point. During the transposition, the discrete arm (Arm-a) is moving the target satellite from the initial pose to the desired pose, while the serpentine arm (Arm-b) is offering auxiliary monitoring for the operation of Arm-a.

### 5.1. The Parameters of Simulation Model

#### 5.1.1. The Parameters of the DSHDASR

**Figure 11:**
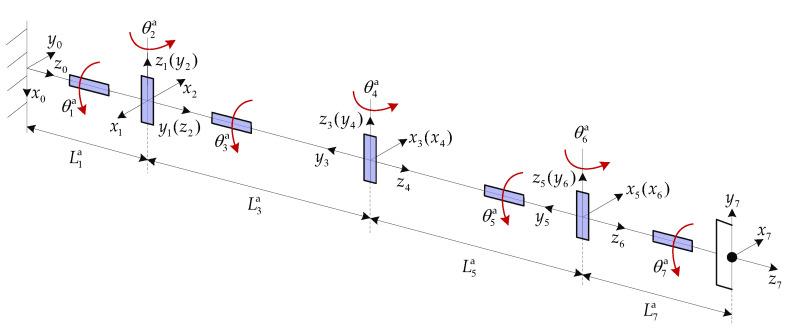
The D-H frames of Arm-a.

The DSHDASR is composed of the base, Arm-a, and Arm-b. Arm-a is a non-offset discrete manipulator with seven degrees of freedom (DoFs). Its D-H frames and parameters are given in Figure 11 and Table 1. The mass parameters are listed in Table 2.

**Figure 12:**
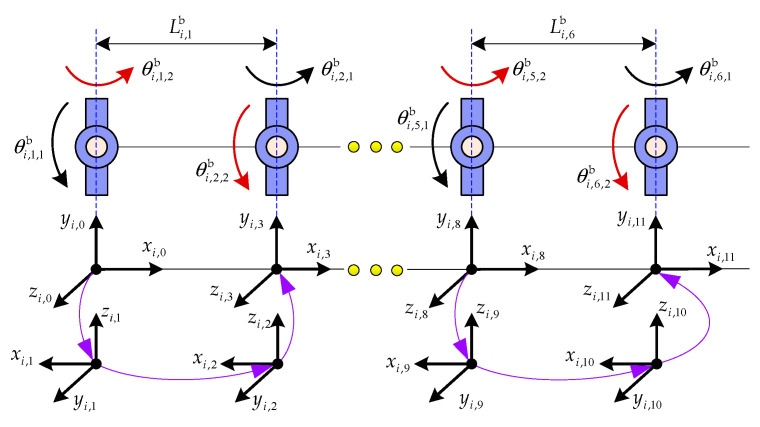
The D-H frames of the *i*th segment for Arm-b.

Arm-b is a segmented serpentine arm with five identical segments. Each segment has six associated universal joints. All segments share an identical mechanical design. The D-H frames and parameters for a representative segment are shown in Figure 12 and provided in Table 3. The length and mass property of each link for Arm-b are given as follows.

(55) $$L_{i,j}^{b}=0.1\;m,\;\;m_{i,j}^{b}=0.1\;\mathrm{kg},\;\;I_{i,j}^{b}=diag\left(0.00011,0.00011,0.0005\right)\;\;\mathrm{kg}\cdot m^{2}$$

The position and attitude (X-Y-Z Euler angle) of the base’s centroid with respect to the inertial frame are as follows.

(56) $$r_{0}=\left[-4.71,\;-3.29,\;70.11\right]\;\mathrm{mm},\;\;\;\Psi_{0}=\left[1.5{}^{\circ},\;0.8{}^{\circ},\;2.1{}^{\circ}\right]$$

The mass and inertia tensor matrix of the base are as follows.

(57) $$m_{0}=3000\;\mathrm{kg},\;\;\;I_{0}=diag\left(3500,\;3500,\;3200\right)\;\mathrm{kg}\cdot m^{2}$$

The installation position and corresponding attitude (X-Y-Z Euler angle) for Arm-a and Arm-b, which are expressed in the frame attached to the base, are given as follows.

(58) $$\left\{\begin{array}{l} b_{0}^{a}=\left[1.3,\;0.585,\;-1.15\right]\mathrm{mm},\;\;\;\Psi_{a}=\left[-180{}^{\circ},\;0{}^{\circ},\;-180{}^{\circ}\right]\\ b_{0}^{b}=\left[1.75,\;0.55,\;1.3\right]\mathrm{mm},\;\;\;\;\;\;\;\;\;\Psi_{b}=\left[0{}^{\circ},\;0{}^{\circ},\;0{}^{\circ}\right] \end{array}\right.$$

#### 5.1.2. The Parameters of the Target Satellite

The mass and inertia tensor matrix of the target satellite are as follows.

(59) $$m_{t}=450\;\mathrm{kg},\;\;\;I_{t}=\left[\begin{array}{ccc} 385.5 & 0.54 & 17.7\\ 0.54 & 327.75 & 19.65\\ 17.7 & 19.65 & 230.4 \end{array}\right]\;\;\;\mathrm{kg}\cdot m^{2}$$

When the target satellite is grasped, the position and attitude (X-Y-Z Euler angle) of the target satellite centroid with respect to the frame attached to the last joint of Arm-a are as follows.

(60) $$r_{\mathrm{et}}=\left[-594.95,\;9.33,\;2662.65\right]\mathrm{mm},\;\;\;\Psi_{t}=\left[-0.0289{}^{\circ},\;-0.0172{}^{\circ},\;138.6069{}^{\circ}\right]$$

### 5.2. The Desired Trajectory of the Target Satellite

To achieve target satellite transposition, the desired trajectory of the target satellite centroid is planned. Following Section 4.2, quintic polynomial interpolation ensured smooth acceleration and deceleration, generating the desired trajectory. In the simulation, the initial and final poses of the target satellite centroid are as follows.

(61) $$\left\{\begin{array}{l} X_{0}=\left[\begin{array}{cccccc} -5.3135 & -0.9097 & -1.2121 & -68.70 & -20.31 & 90.76 \end{array}\right]\\ X_{f}=\left[\begin{array}{cccccc} -3.8366 & 2.8592 & -2.0086 & -121.49 & -2.88 & 101.27 \end{array}\right] \end{array}\right.$$

**Figure 13:**
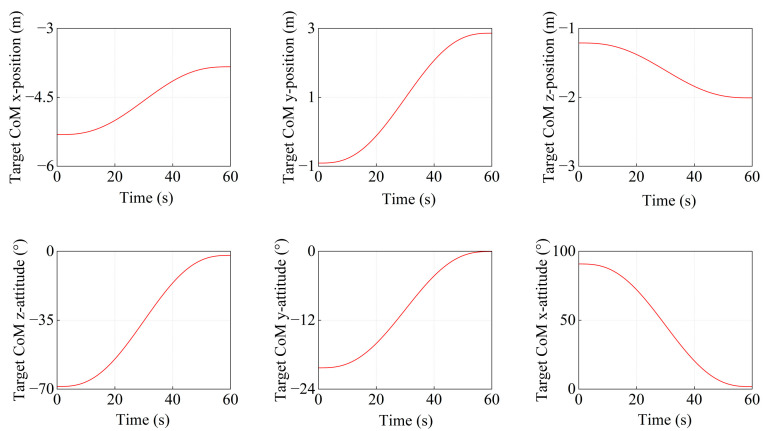
The variation curves of the pose for the target satellite centroid.

Substituting Equation (61) into Equation (17), the variations of position and attitude for the centroid of the target satellite are obtained, as shown in Figure 13.

### 5.3. The Simulation for the Pose Stabilization of the Combined System Base

#### 5.3.1. The Simulation Results for the Proposed Method

At the initial moment, the joint angles for Arm-a and Arm-b are set as follows.

(62) $$\begin{array}{l} \Phi_{a}=\left[1.7486,\;1.2895,\;-0.1394,\;1.1473,\;-0.3021,\;-0.9054,\;-0.1692\right]\mathrm{rad}\\ \Phi_{b}=\left[-0.011,\;0.006,\;-0.0046,\;0.0503,\;-0.0072,\;-0.0059,\;-0.0227,\;0.0263,\;0.0217,\;0.1402\right]\mathrm{rad} \end{array}$$

**Figure 14:**
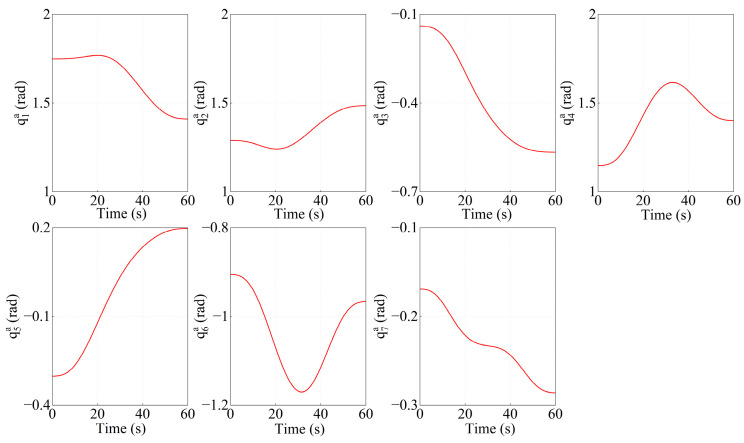
The variation curves of joint angles for Arm-a.

**Figure 15:**
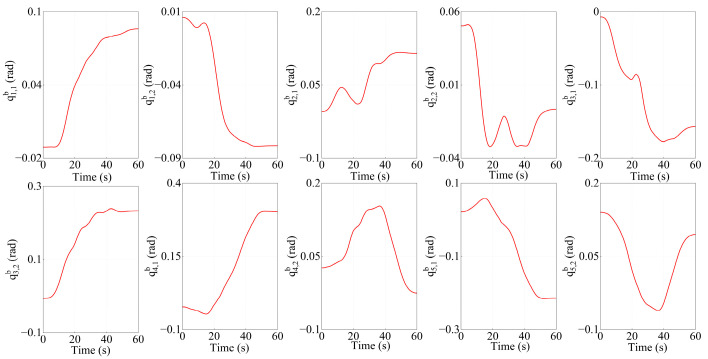
The variation curves of joint angles for Arm-b.

From the target centroid trajectory (as indicated in Figure 13), the desired trajectories of the Arm-a tip and Arm-b tip can be obtained. Furthermore, the joint angles of both arms are calculated by the inverse kinematics solution. The variation curves of the joint angles are shown in Figure 14 and Figure 15, respectively.

In the simulation, the proportional and derivative coefficient matrices corresponding to the position and attitude of the base are set as follows.

(63) $$\begin{array}{ll} K_{\mathrm{pp}}=\left[\begin{array}{ccc} 12000 & 0 & 0\\ 0 & 19000 & 0\\ 0 & 0 & 14000 \end{array}\right], & K_{\mathrm{vv}}=\left[\begin{array}{ccc} 300 & 0 & 0\\ 0 & 400 & 0\\ 0 & 0 & 400 \end{array}\right]\\ K_{p\varphi }=\left[\begin{array}{ccc} 6400 & 0 & 0\\ 0 & 5600 & 0\\ 0 & 0 & 6400 \end{array}\right], & K_{v\omega }=\left[\begin{array}{ccc} 160 & 0 & 0\\ 0 & 320 & 0\\ 0 & 0 & 320 \end{array}\right] \end{array}$$

Here, external disturbance forces and torques are introduced to simulate external environment influences, such as solar radiation pressure. The forces and torques are set as follows.

(64) $$F_{r}={\left[\begin{array}{ccc} -0.05, & 0.22, & -0.33 \end{array}\right]}^{T}N,\;\;\;\tau_{r}={\left[\begin{array}{ccc} 0.076, & -0.11, & 0.047 \end{array}\right]}^{T}N\cdot m^{-1}$$

**Figure 16:**
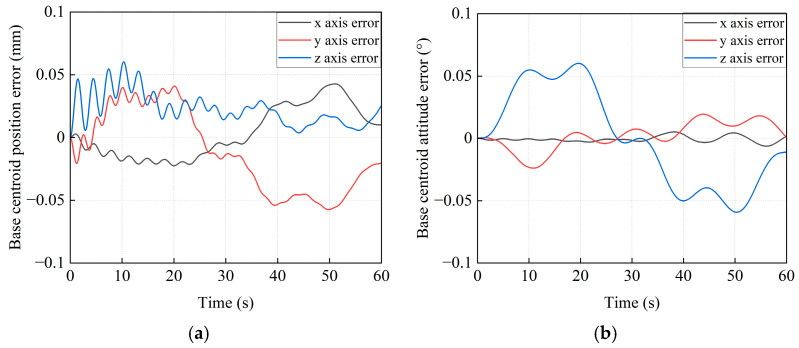
The pose error curves for the base centroid of the combined system. (**a**) The position error; (**b**) The attitude error.

Figure 16 illustrates the base’s pose variations during transposition under the proposed control method. The maximum position deviations of the base centroid are 0.043 mm, −0.057 mm, and 0.06 mm along the *x*_I_, *y*_I_, and *z*_I_ axes (see Figure 16a), respectively. The maximum deviations of attitude (X-Y-Z Euler angles, Figure 16b) are −0.006°, −0.024°, and 0.06°, respectively. The simulation results indicate that pose error is maintained within 0.1 mm and 0.1° by the proposed method, whose accuracy meets the engineering requirements.

**Figure 17:**
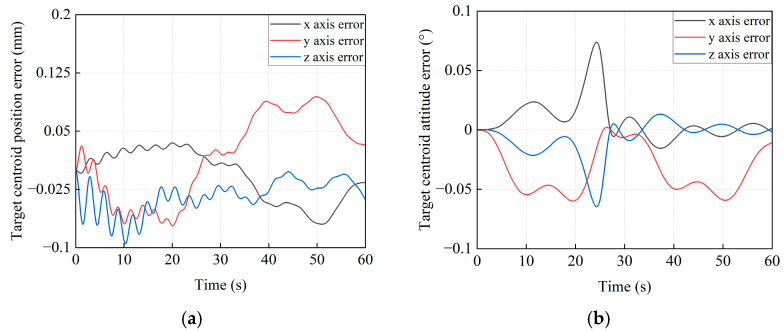
The trajectory tracking error curves of the target satellite. (**a**) The position tracking error. (**b**) The attitude tracking error.

Due to base pose deviations, the actual trajectory tracking of the target satellite could deviate from the desired trajectory. The trajectory tracking errors are shown in Figure 17. It can be observed that the maximum position errors of the target satellite centroid are −0.07 mm, 0.094 mm, and −0.094 mm (Figure 17a) corresponding to the *x*_I_, *y*_I_, and *z*_I_ axes, while the maximum attitude errors during this process are 0.083°, –0.06°, and −0.072° (Figure 17b), respectively. Both position and attitude errors are limited within 0.1 mm and 0.1°, which further validate the correctness of the proposed method.

**Figure 18:**
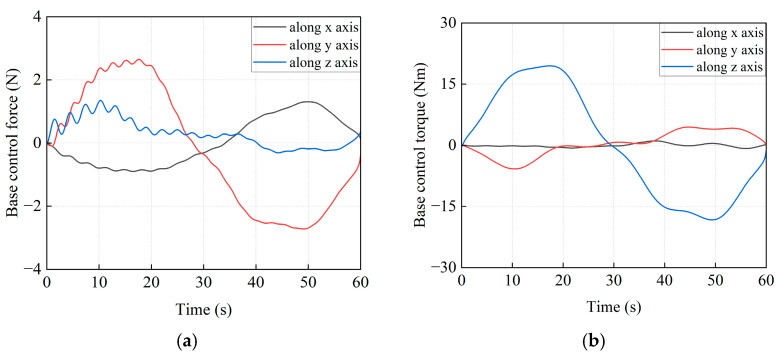
The curves of the control forces and torques. (**a**) The control forces on the base of the combined system. (**b**) The control torques on the base of the combined system.

**Figure 19:**
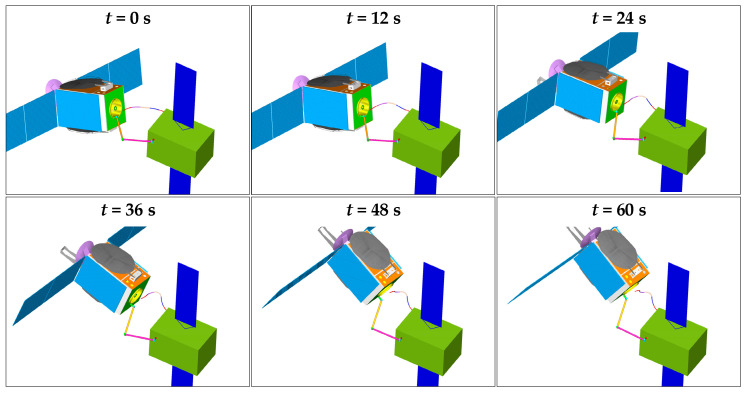
The different states of the combined system in the simulation.

The control forces and torques, which are applied to the base, are shown in Figure 18. They can be generated by thrusters and reaction wheels. Figure 19 shows the 3D simulation models at the following milestones: t = 0 s, 12 s, 24 s, 36 s, 48 s, and 60 s.

#### 5.3.2. The Simulation Results for the PD Control Method

**Figure 20:**
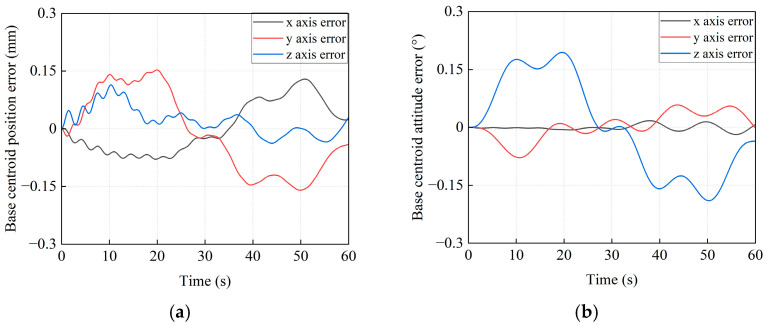
The pose error curves for the base centroid of the combined system by the PD control method. (**a**) The position error of the base centroid. (**b**) The attitude error of the base centroid.

**Figure 21:**
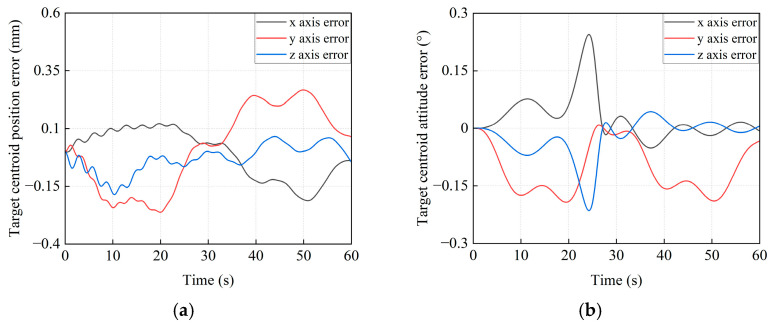
The trajectory tracking error curves of the target satellite centroid by the PD control method. (**a**) The position tracking error. (**b**) The attitude tracking error.

To further validate the effectiveness of the proposed control strategy in stabilizing the base’s pose, a comparative simulation is conducted by using the PD control without feedforward compensation. When the traditional PD control method is applied, the pose variations for the base centroid of the combined system are given in Figure 20. As shown in Figure 20a, the simulation results indicate that the maximum position deviations of the base centroid along the *x*_I_, *y*_I_, and *z*_I_ axes are 0.1289 mm, −0.1599 mm, and 0.1139 mm, respectively. It can be seen that all errors exceeded 0.1 mm. The maximum attitude deviations of the base centroid are −0.0186°, −0.0785°, and 0.1945° (see Figure 20b), respectively. Notably, the *z*_I_ axis error exceeds 0.1°. In addition, the trajectory tracking deviations of the target satellite centroid are shown in Figure 21. It can be concluded that the maximum position tracking errors of the target satellite centroid are −0.2121 mm, 0.2665 mm, and −0.1857 mm, respectively. Meanwhile, they are 0.2442°, 0.1926°, and −0.2142° for the attitude tracking errors. All position errors and attitude errors are more than 0.1 mm and 0.1°, respectively. These results indirectly show the superior control accuracy of the proposed method over the traditional PD control method.

#### 5.3.3. The Simulation Results for the Backstepping Method

**Figure 22:**
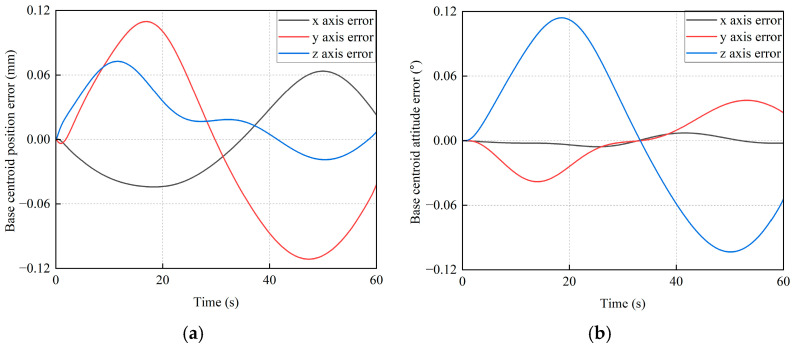
The pose control error curves of the base by the Backstepping method. (**a**) The position error of the base centroid. (**b**) The attitude error of the base centroid.

**Figure 23:**
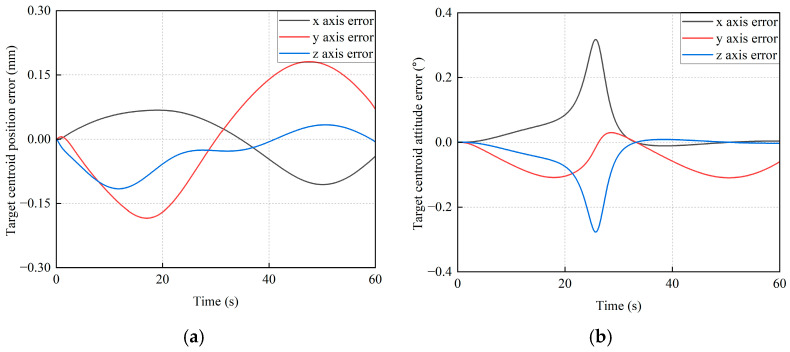
The trajectory tracking error curves of the base by the Backstepping method. (**a**) The position tracking error. (**b**) The attitude tracking error.

In order to further verify the advantages of the proposed method, another comparison method (i.e., the Backstepping method) is implemented. The pose control errors of the base and the pose tracking error of the target satellite are shown in Figure 22 and Figure 23, respectively.

From Figure 22, it can be seen that the maximum position and attitude control errors of the base along *x*_I_, *y*_I_, and *z*_I_ axes are 0.0636 mm, 0.1114 mm, 0.0724 mm, 0.0072°, 0.0381°, and 0.1141°, respectively. As given in Figure 23, the maximum position and attitude tracking errors of the target satellite along *x*_I_, *y*_I_, and *z*_I_ axes are 0.1060 mm, 0.1840 mm, 0.1158 mm, 0.3176°, 0.1093°, and 0.2774°, respectively.

### 5.4. Simulation Results Analysis

The simulation results for the PD control method, the Backstepping method, and the proposed method are listed in Table 4.

According to the simulation results, the position control accuracy for the base of the combined system based on the proposed method is improved by 66.64%, 64.35%, and 47.32% along the *x*_I_, *y*_I_, and *z*_I_ axes, respectively, compared with the PD control method. The attitude control accuracy of the base is improved by 66.13%, 69.55%, and 69.05%, respectively. Due to the dynamic coupling between the combined system’s base and the two manipulators, it will lead to the occurrence of a trajectory tracking error for the target satellite centroid. The proposed method achieves 67.04%, 64.62%, and 48.90% improvement in position tracking accuracy, compared with the PD control method. Additionally, they are 69.78%, 68.90%, and 69.84% for the attitude tracking accuracy.

Compared with the Backstepping method, the accuracy in both position and attitude control of the base are improved by 32.39%, 48.83%, 17.12%, 12.50%, 37.27%, and 47.24%, respectively. The position tracking accuracy of the target satellite are improved by 34.06%, 48.75%, and 18.05%, respectively. They are 76.76%, 45.20% and 76.71% for attitude tracking accuracy.

As a result, it can be concluded that the proposed method significantly improves the pose control precision of the base and the pose tracking accuracy of the target satellite. The effectiveness of the proposed method is thoroughly verified.

Regarding the comparison with the Backstepping method, it should be noted that the Backstepping controller is introduced in this study as a base stabilization strategy, with the same control objective as the conventional PD controller, namely suppressing the base disturbances induced by the manipulator and target satellite motion. The Backstepping method is not designed to directly optimize the attitude tracking performance of the target satellite. For a fair comparison, both controllers are tuned to achieve comparable settling times. In addition, relatively conservative virtual control gains are adopted for the Backstepping controller to ensure stability under strong dynamic coupling and large payload conditions.

In addition, the sensor noise is accounted for by incorporating model parameter uncertainty. The simulations are performed for parameter uncertainty levels of 2%, 5%, and 10%, respectively, to evaluate each scenario. The simulation results are listed in Table 5. It can be concluded that a larger parameter uncertainty leads to greater control errors. Furthermore, with a parameter uncertainty of 15%, the maximum position control error of the base is less than 0.09 mm, and the attitude error is below 0.11°. Meanwhile, for target satellite transposition, the position tracking error is less than 0.15 mm, and the attitude error remains under 0.12°.

In this analysis, the parametric uncertainty scenarios listed in Table 5 primarily focus on variations in the system mass and inertial properties. It is further clarified that sensor noise effects, which introduce measurement errors in the feedback loop and in the estimation of external disturbance forces and torques, are not explicitly modeled as stochastic processes, but are instead implicitly reflected through bounded uncertainty and equivalent disturbance terms within the adopted parametric uncertainty framework.

## 6. Discussion

A more comprehensive discussion of the challenges and simplifications associated with real-world implementation is given, including time delay, computational cost, and actuator saturation. Regarding the time delay issue, system delays from sensor sampling, communication, and computation could introduce phase errors, particularly degrading the performance of the model-dependent feedforward compensation. This would place a greater burden on the robustness of the PD feedback loop. We will study some strategies like predictive filtering or high-speed data protocols to address this issue. On the problem of computational cost, the real-time execution of the recursive Newton–Euler algorithm for feedforward dynamics, while efficient (*o*(*n*)), must be rigorously validated on representative flight processor hardware. About the actuator saturation, the physical limits of the thrusters and reaction wheels are a critical constraint. Each actuator requires integrating an actuator saturation management block and a control allocation strategy to dynamically distribute torque between actuators, preventing performance degradation or instability when commanded values exceed physical limits. It should be noted that the implementation-related aspects, including computation time per Newton–Euler recursion iteration, delay tolerance, and actuator saturation limits, are intended as qualitative considerations derived from simulation analysis. These discussions aim to provide preliminary insights into the practical feasibility of the proposed control strategy, rather than to report quantitatively validated hardware-level performance metrics. A detailed quantitative evaluation of these aspects requires hardware implementation or hardware-in-the-loop experiments, which are beyond the scope of the present study and will be addressed in future studies.

The base pose stabilization control method proposed in this study has so far been validated only in a simulation environment, and the analysis results are obtained under a set of idealized modeling assumptions. Specifically, the mass and geometric parameters of the system are assumed to be accurately known, while non-ideal factors such as joint friction, structural flexibility, and mechanical backlash are neglected in the simulation. Consequently, the simulation results mainly reflect the control performance of the proposed method in suppressing base dynamic coupling disturbances induced by large payload motions under ideal conditions. The advantage of simulation-based studies lies in their ability to evaluate the effectiveness and stability of the proposed strategy in a controllable and repeatable environment, which facilitates the investigation of system dynamics and the underlying control mechanisms. However, in practical physical implementations, factors such as sensor noise, actuator constraints, computational delays, and modeling inaccuracies may adversely affect control performance. To address these issues, future work will incorporate parameter uncertainties, joint friction, and structural flexibility, and will further assess the engineering applicability of the proposed method through higher-fidelity simulations or experimental platforms.

## 7. Conclusions

This paper proposed a pose stabilization algorithm based on feedforward compensation PD control to stabilize the base of the combined system during target satellite transposition. The overall transposition mission was analyzed to define the roles of each manipulator. Then, a dynamics model of the combined system was established to calculate the feedforward compensation term. A pose stabilization strategy combing feedforward compensation and PD control was developed to suppress disturbances to the base. Finally, to verify the proposed method, a co-simulation model was established byMatlab2016 and Adams2018 software. Comparative simulations were conducted for the proposed method and the traditional method. By incorporating the reaction forces and torques exerted on the base as feedforward compensation terms in the control law, the proposed method effectively mitigates the strong dynamic coupling effects during the target satellite transposition process. As a result, the errors of the base’s position and attitude are reduced significantly, and the trajectory tracking accuracy is improved for the transposition operation. Simulation results indicate that the proposed method exhibits good applicability for base stabilization control under large payload conditions, providing a feasible solution for maintaining base pose stability during the target satellite transposition process.

The current research only focuses on pose stabilization of the base for the combined system that is composed of a heterogeneous multi-arm space robot and a target satellite. The application of these methods for heterogeneous multi-arm systems has not been explored. Its dynamic recursive formulation does not account for joint friction or mechanical clearance. Furthermore, the robustness validation in this study is restricted to deterministic bounded-uncertainty scenarios rather than statistical characterization. Future work will incorporate stochastic analysis methods, such as Monte Carlo simulations, to statistically evaluate the control performance under random measurement noise and time-varying disturbances. In addition, the minimum energy optimization for target satellite transposition is also not considered in this paper. In the future, we will strive to do research on these topics.

## Figures and Tables

**Table 1:** The D-H parameters of Arm-a.

Link *i*	$\theta_{i}$ (°)	$\alpha_{i}$ (°)	$a_{i}$ (m)	$d_{i}$ (m)
1	−90	90	0	$L_{1}^{a}=0.255$
2	180	90	0	0
3	0	−90	0	$L_{3}^{a}=1.9$
4	0	90	0	0
5	0	−90	0	$L_{5}^{a}=1.9$
6	0	90	0	0
7	0	0	0	$L_{7}^{a}=0.255$

**Table 2:** The mass parameters of Arm-a.

Link		$B_{1}^{a}$	$B_{2}^{a}$	$B_{3}^{a}$	$B_{4}^{a}$	$B_{5}^{a}$	$B_{6}^{a}$	$B_{7}^{a}$
Mass (kg)		4.24	4.24	8.675	4.24	8.675	4.24	4.24
*^i^**a**_i_* (m)	*x*	0	0	0	0	0	0	0
	*y*	0	0	0	0	0	0	0
	*z*	0.1275	0	0.95	0	0.95	0	0.1275
*^i^**b**_i_* (m)	*x*	0	0	0	0	0	0	0
	*y*	0	0	0	0	0	0	0
	*z*	0.1275	0	0.95	0	0.95	0	0.1275
*^i^**I**_i_* (kg·m^2^)	*I_xx_*	0.018	0.018	3.846	0.018	3.846	0.018	0.0055
	*I_yy_*	0.018	0.018	3.846	0.018	3.846	0.018	0.0055
	*I_zz_*	0.0075	0.0075	0.393	0.0075	0.393	0.0075	0.0065
	*I_xy_*	0	0	0	0	0	0	0
	*I_xz_*	0	0	0	0	0	0	0
	*I_yz_*	0	0	0	0	0	0	0

**Table 3:** The D-H parameters of the *i*th segment for Arm-b.

Segment	Rotation Axis	$\theta_{i,j,k}$ (°)	$\alpha_{i,j,k}$ (°)	$a_{i,j,k}$ (m)	$d_{i,j,k}$ (m)
*i*	1	0	90	0	0
	2	0	0	$L_{i,1}^{b}=0.1$	0
	3	0	−90	0	0
	4	0	0	$L_{i,2}^{b}=0.1$	0
	⋮	⋮	⋮	⋮	⋮
	9	0	90	0	0
	10	0	0	$L_{i,5}^{b}=0.1$	0
	11	0	−90	0	0
	12	0	0	$L_{i,6}^{b}=0.1$	0

**Table 4:** The simulation results of the two methods.

Item	Coordinate Axis	PD Control	Backstepping	Proposed Method	Improvement over PD	Improvement over Backstepping
Position error of the base	*x*_I_	0.1289	0.0636	0.0430	66.64%	32.39%
	*y*_I_	−0.1599	0.1114	−0.0570	64.35%	48.83%
	*z*_I_	0.1139	0.0724	0.0600	47.32%	17.12%
Attitude error of the base	*x*_I_	−0.0186	0.0072	−0.0063	66.13%	12.50%
	*y*_I_	−0.0785	0.0381	−0.0239	69.55%	37.27%
	*z*_I_	0.1945	0.1141	0.0602	69.05%	47.24%
Position tracking error	*x*_I_	−0.2121	0.1060	−0.0699	67.04%	34.06%
	*y*_I_	0.2665	0.1840	0.0943	64.62%	48.75%
	*z*_I_	−0.1857	0.1158	−0.0949	48.90%	18.05%
Target satellite attitude tracking error	*x*_I_	0.2442	0.3176	0.0738	69.78%	76.76%
	*y*_I_	−0.1926	−0.1093	−0.0599	68.90%	45.20%
	*z*_I_	−0.2142	0.2774	−0.0646	69.84%	76.71%

**Table 5:** The simulation results under the condition of parameter uncertainty.

Item	Coordinate Axis	2%	5%	10%	15%
Position error of the base	*x*_I_	0.0461	0.0510	0.0589	0.0664
	*y*_I_	−0.0614	−0.0672	−0.0766	−0.0854
	*z*_I_	0.0624	0.0655	0.0704	0.0750
Attitude error of the base	*x*_I_	−0.0068	−0.0075	−0.0086	−0.0096
	*y*_I_	−0.0261	−0.0291	−0.0341	−0.0388
	*z*_I_	0.0651	0.0728	0.0849	0.0963
Position tracking error of the target satellite	*x*_I_	−0.0755	−0.0836	−0.0966	−0.1089
	*y*_I_	0.1010	0.1110	0.1261	0.1413
	*z*_I_	−0.0985	−0.1037	−0.1120	−0.1198
Attitude tracking error of the target satellite	*x*_I_	0.0802	0.0895	0.1045	0.1187
	*y*_I_	−0.0649	−0.0724	−0.0843	−0.0956
	*z*_I_	−0.0702	−0.0784	−0.0916	−0.1041

## Data Availability

The original contributions presented in this study are included in the article. Further inquiries can be directed to the corresponding author.
